# Chemical perturbation reveals a cytoskeletal–trafficking vulnerability in *Haemonchus contortus*

**DOI:** 10.3389/fphar.2026.1866526

**Published:** 2026-08-14

**Authors:** Aya C. Taki, Nghi Nguyen, Tao Wang, Joseph J. Byrne, Anson V. Koehler, Harrison T. Shanley, Ching-Seng Ang, Jennifer Keiser, Cécile Häberli, Brad E. Sleebs, Robin B. Gasser

**Affiliations:** 1 Department of Veterinary Biosciences, Melbourne Veterinary School, Faculty of Science, The University of Melbourne, Parkville, VIC, Australia; 2 Walter and Eliza Hall Institute of Medical Research, Parkville, VIC, Australia; 3 Melbourne Mass Spectrometry and Proteomics Facility, The Bio21 Molecular Science and Biotechnology Institute, The University of Melbourne, Parkville, VIC, Australia; 4 Medical Parasitology and Infection Biology, Swiss Tropical and Public Health Institute, Allschwil, Switzerland; 5 University of Basel, Basel, Switzerland

**Keywords:** anthelmintic discovery, chemical perturbation, cytoskeleton, intracellular trafficking, parasitic nematodes, proteome integral solubility alteration (PISA)

## Abstract

The control of parasitic nematodes of humans and animals remains heavily dependent on a limited number of anthelmintic drug classes, and resistance to major classes is now widespread. Although phenotypic screening readily identifies compounds that impair worm motility or development, the intrinsic biological processes underlying chemical sensitivity in parasitic nematodes remain poorly defined. Here, we identified a hit compound with a pyridyl scaffold from a phenotypic screen against the model parasitic nematode, *Haemonchus contortus*, and using structure–activity optimisation we generated a potent chemical probe, WEHI-684. To uncover protein networks associated with the mechanism of action, thermal proteome profiling and time-resolved quantitative proteomics interrogated WEHI-684-induced perturbations in *H*. *contortus*. Across larval and adult stages of this major parasite of livestock, proteome integral solubility alteration (PISA) profiling revealed reproducible alterations in proteins associated with cytoskeletal organisation and intracellular trafficking, including actin- and motor-related components. Complementary quantitative proteomics identified induction of an aspartyl protease and suppression of secretory CAP family proteins. Integrated analysis of these datasets supports a model in which chemical perturbation of cytoskeletal and trafficking proteins is associated with secondary modulation of proteolytic pathways, coinciding with rapid impairment of motility. These findings indicate that linked structural and proteolytic responses contribute to chemical sensitivity in *H. contortus* and demonstrate how integrative proteomics can resolve organism-level responses to chemical perturbation beyond single-target paradigms.

## 1 Introduction

Parasitic nematodes impose a major and persistent burden on global human and animal health, with gastrointestinal and tissue-dwelling species responsible for substantial morbidity, production losses and economic cost (Selzer and Epe, 2021; Kaminsky and Mäser, 2025). Control strategies remain heavily dependent on a limited number of anthelmintic drug classes, including benzimidazoles, macrocyclic lactones and imidazothiazoles (Lanusse et al., 2018; Jiao et al., 2020). However, resistance to all major classes is now widespread in livestock nematodes and increasingly reported in human helminths (Geerts and Gryseels, 2001; Kotze et al., 2014; Charlier et al., 2023; Coffeng et al., 2024). In many production systems, multidrug-resistant nematode populations are established, reducing therapeutic efficacy and compromising long-term control strategies (Charlier et al., 2022). Despite this growing resistance crisis, the pace of discovery of new anthelmintic classes has been slow, and relatively few mechanistically distinct compounds have advanced beyond early development (Zajíčková et al., 2019; Nixon et al., 2020). A fundamental limitation in anthelmintic innovation is the incomplete understanding of the intrinsic biological dependencies that render parasitic nematodes susceptible to chemical perturbation (Partridge et al., 2020; Shanley et al., 2025).

Historically, anthelmintic discovery has been guided by single-target paradigms, often extrapolated from pharmacological insights in model organisms or other metazoans (Geary and Thompson, 2001; Holden-Dye and Walker, 2014; Burns et al., 2015; Hahnel et al., 2020; Roy, 2025). While this approach has yielded successful drugs targeting ion channels or microtubule assembly (Driscoll et al., 1989; Fleming et al., 1997; Martin et al., 2005; Saunders et al., 2013), for instance, it may overlook higher-order cellular interdependencies that are particularly important in parasitic helminths (Roy, 2025). Phenotypic screening provides a route to identifying compounds that impair viability, development, reproduction or motility (Jiao et al., 2020; Herath et al., 2022), yet translating phenotypic hits into mechanistic understanding remains challenging (Marhöfer et al., 2025). Consequently, although phenotypic assays readily identify compounds that impair parasite viability or function, they do not inherently reveal which cellular systems confer chemical sensitivity or whether shared vulnerabilities exist within or across developmental stages (Moffat et al., 2017; Roy, 2025).

Motility is central to nematode survival and pathogenicity (Lok, 2016). Coordinated movement enables feeding, tissue migration and host interaction, and depends on tightly regulated actin–myosin dynamics, motor protein activity and neuromuscular signalling (Gieseler et al., 2017). Beyond contractile function, cytoskeletal organisation is intimately linked to vesicle trafficking, membrane remodelling and secretion (Yang et al., 2020). Through these trafficking functions, the cytoskeleton contributes not only to movement but also to intracellular transport processes that sustain parasite physiology. In multicellular helminths, intracellular transport pathways support the trafficking and secretion of parasite-derived proteins that contribute to development and host adaptation (Moreno et al., 2021; Cortés et al., 2025). Disruption of cytoskeletal architecture can therefore propagate through trafficking networks and protein turnover pathways, potentially destabilising cellular homeostasis (Monastyrska et al., 2009). Proteolytic systems, including cysteine and aspartyl proteases, further contribute to nutrient acquisition, tissue invasion and stress adaptation in parasitic nematodes (Yang et al., 2023; Lin and Siddique, 2024). However, whether structural and proteolytic processes constitute a coordinated vulnerability in parasitic nematodes has not been systematically examined (Yang et al., 2020).

Recent advances in thermal proteome profiling and quantitative mass spectrometry (Gaetani et al., 2019; Perrin et al., 2020; Batth et al., 2024) now enable proteome-wide interrogation of compound-induced perturbations. Thermal stability shifts provide a means to detect direct or proximal protein engagement in complex lysates (Mateus et al., 2020; Shanley et al., 2025), while time-resolved quantitative proteomics can resolve temporal changes in protein abundance following chemical perturbation (Baxi et al., 2023). When integrated with phenotypic screening and structure–activity optimisation, these approaches allow small molecules to serve not only as candidate therapeutics but also as chemical probes for dissecting cellular dependencies (Pasquer et al., 2020). Rather than focusing solely on identifying individual molecular targets, such integrative strategies can illuminate network-level perturbations that expose intrinsic biological vulnerabilities (Maity et al., 2024).

In this study, we employ an optimised pyridyl scaffold, initially identified through phenotypic screening of the Maybridge HitFinder compound library (Taki et al., 2022), as a chemical probe to investigate molecular responses to chemical perturbation in *H. contortus*, a tractable model parasitic nematode (Ma et al., 2020; Zheng et al., 2025). Using thermal proteome profiling and time-resolved quantitative proteomics across larval and adult stages, we sought to define the cellular processes most susceptible to chemical perturbation and to determine whether convergent network-level perturbations emerge across developmental contexts.

## 2 Results

### 2.1 Structure–activity optimisation generates potent probes for mechanistic interrogation

**FIGURE 1:**
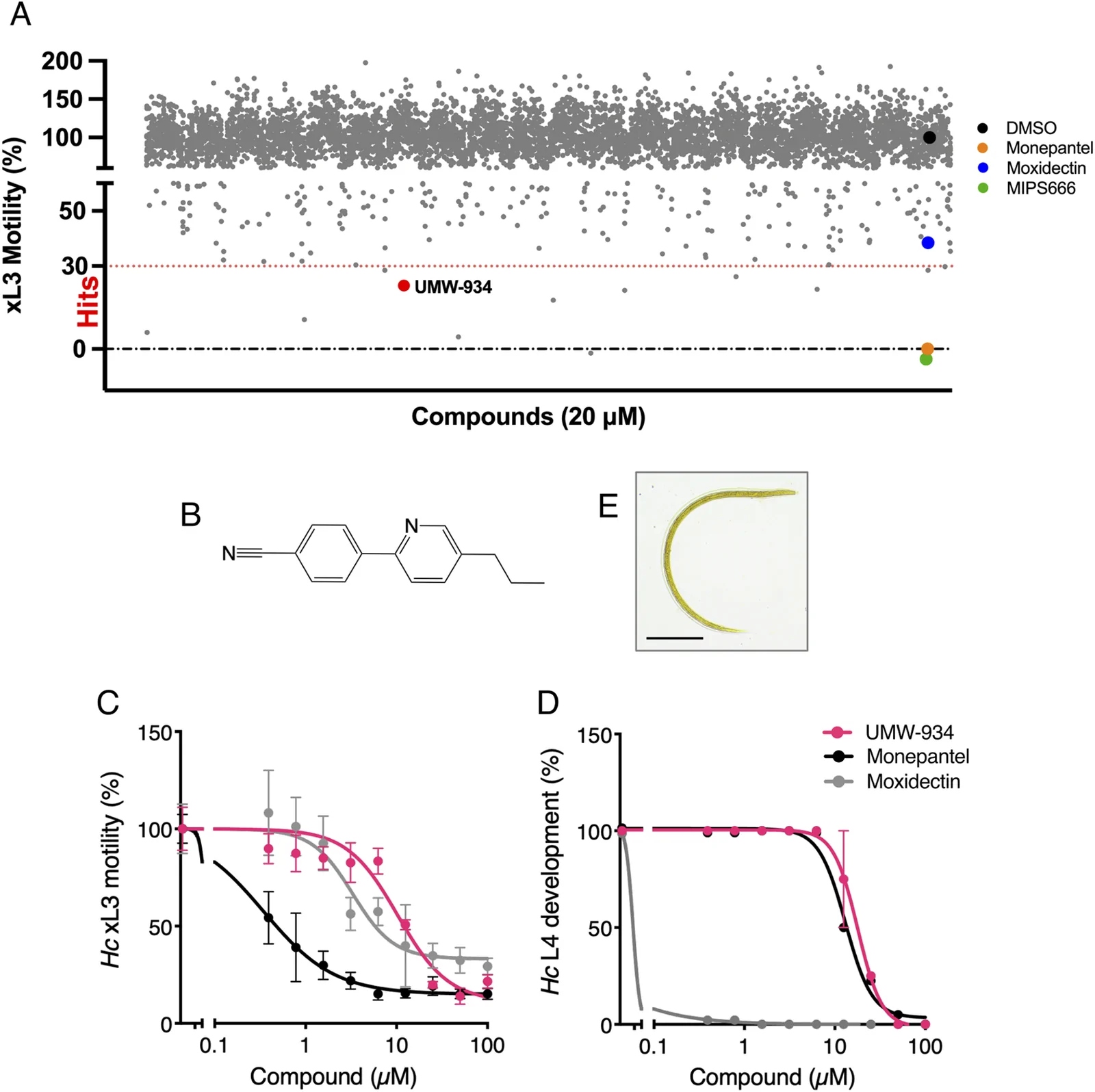
Identification and primary biological characterisation of UMW-934 against *Haemonchus contortus*. **(A)** High-throughput phenotypic screening of 14,400 small molecules against exsheathed third-stage larvae (xL3s) of *H. contortus*. Compounds were evaluated for inhibition of larval motility under standard culture conditions. Molecules causing ≥70% reduction in motility relative to vehicle (0.25% DMSO) controls were classified as hits. UMW-934 was prioritised for further evaluation based on reproducible activity. **(B)** Chemical structure of UMW-934, a pyridyl benzonitrile scaffold identified from the primary screen. **(C)** Concentration–response curve for inhibition of xL3 motility following 168 h incubation at 38°C under 10% CO_2_ and >90% relative humidity. Motility was quantified using a standardised scoring system and normalised to vehicle controls. Data represent mean ± SEM from three independent experiments performed in triplicate wells. IC_50_ values were estimated using a variable-slope four-parameter non-linear regression model. **(D)** Inhibition of larval development (xL3 to L4 transition) following 168 h exposure to UMW-934. Developmental progression was assessed microscopically, and the minimum inhibitory concentration (MIC) corresponding to complete inhibition of development was recorded. **(E)** A representative image of a larva displaying a curved phenotype following 168 h exposure to UMW-934 (scale: 100 µm). Data represent mean ± SEM from three independent experiments.

**FIGURE 2:**
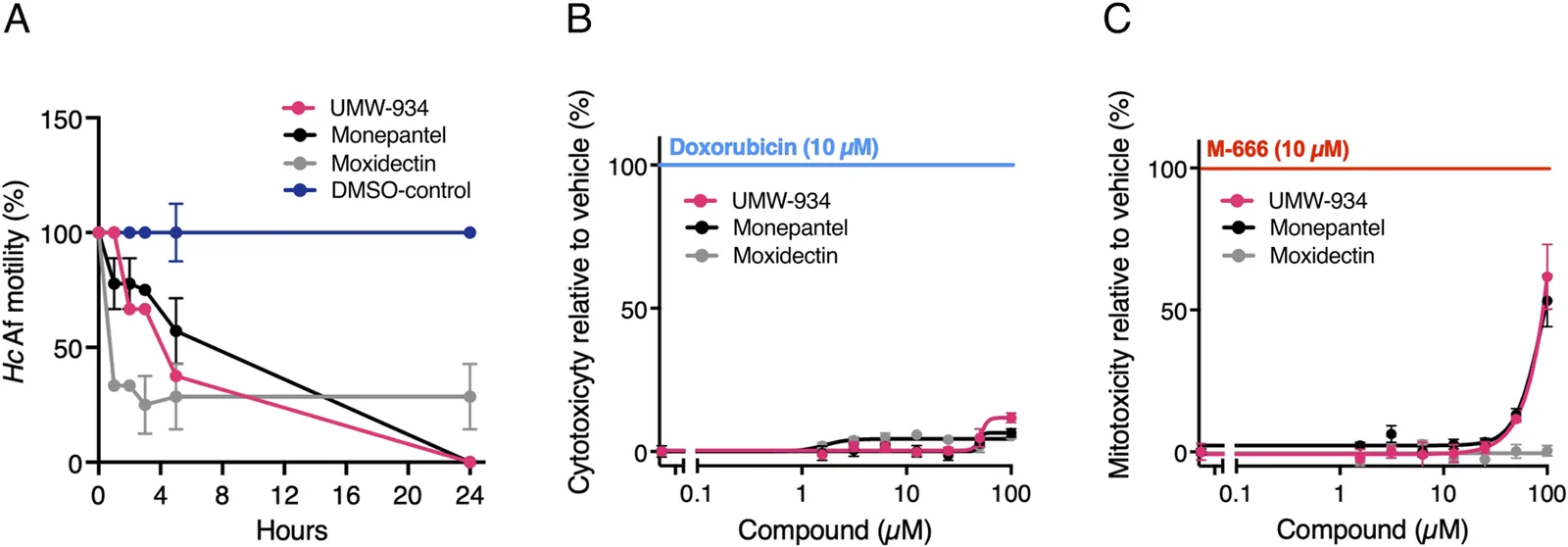
Adult-stage activity and *in vitro* toxicity assessment of UMW-934. **(A)** Inhibition of motility of adult female *Haemonchus contortus* following 24 h exposure to UMW-934 at 100 µM. Motility was assessed using a standardised scoring system and normalised to vehicle (0.25% DMSO) controls. Data represent mean ± SD from triplicate wells. **(B)** Cytotoxicity of UMW-934 toward HepG2 human hepatoma cells following 48 h exposure. Cell viability was determined by crystal violet staining and normalised to vehicle controls. **(C)** Assessment of mitochondrial toxicity in HepG2 cells cultured in galactose-containing medium to promote oxidative phosphorylation dependency. Cells were exposed to UMW-934 for 48 h under serum-starved conditions prior to viability assessment. CC_50_ and MC_50_ values were calculated using a variable-slope four-parameter non-linear regression model. Data represent mean ± SEM from three independent experiments.

An initial pyridyl benzonitrile scaffold **(UMW-934)** was prioritised from an unbiased phenotypic screen of 14,400 small molecules based on its reproducible inhibition of motility in exsheathed third-stage larvae (xL3s) of *H. contortus* (Figures 1A,B). Subsequent validation confirmed concentration-dependent inhibition of larval motility and development, as well as activity against adult females (Figures 1C–E, 2A). Importantly, the scaffold displayed limited cytotoxicity and mitotoxicity in HepG2 cells, supporting further optimisation (Figures 2B,C).

**FIGURE 3:**
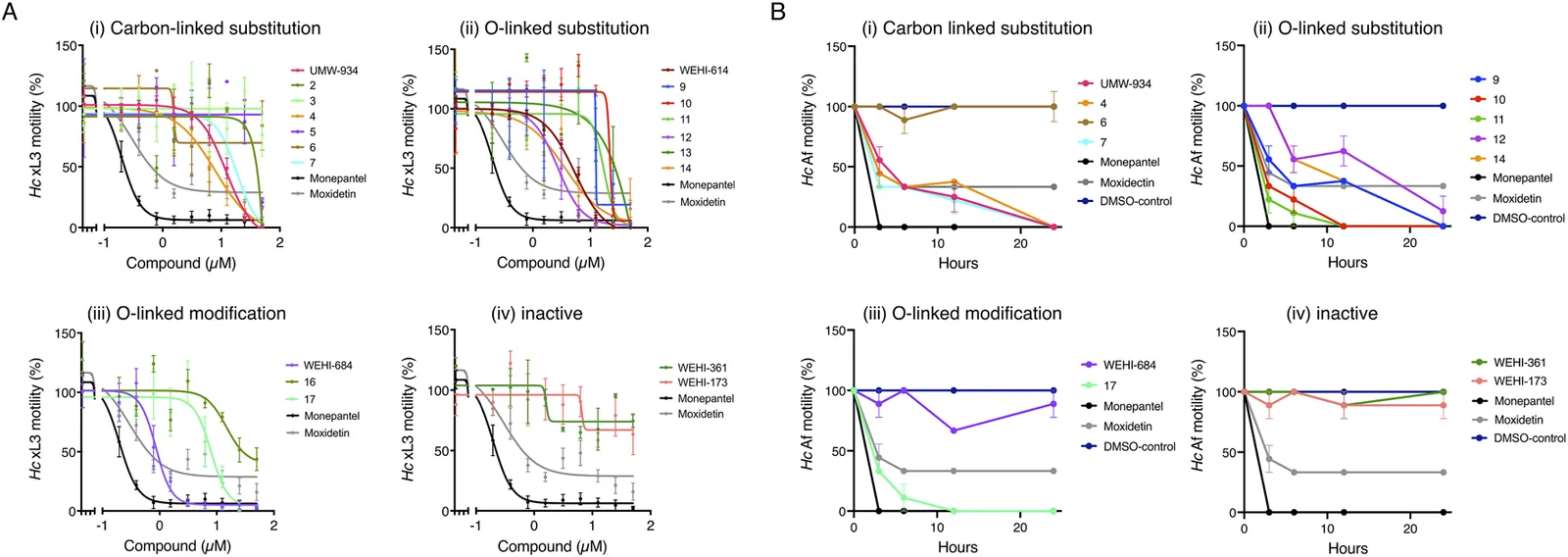
Potency of optimised UMW-934 analogues against larval and adult stages of *Haemonchus contortus*. *In vitro* inhibition of motility of **(A)** larvae following 168 h exposure or **(B)** adult female *H. contortus* following 24 h exposure to UMW-934 and selected active analogues, together with two inactive analogues (**3** and **6**; panel (iv)). Compounds were grouped according to structural modification: (i) carbon linked substitutions, (ii) O-linked substitutions and (iii) O-linked modifications (see Table 1). Monepantel and moxidectin were included as active reference compounds, and vehicle (0.25% DMSO) served as the negative control. For adult female, motility scores were normalised to vehicle controls and expressed as percentage inhibition. Data represent mean ± SEM (larvae) or SD (adult female) from a representative experiment conducted in triplicate.

To enhance potency and enable mechanistic interrogation, we undertook systematic structure–activity optimisation of the scaffold (Table 1; Figure 3A). We found that the 4-nitrle aryl motif was refractory to alteration, and therefore we focused on changes to the 3-position of the pyridyl ring. Modifications to the 3-alkyl substituent on the pyridyl ring revealed defined chain-length constraints, with the 3-ethyl (analogue **2**, IC_50_ 30 µM) and 3-pentyl (analogue **5**, IC_50_ 27 µM) substituents were ∼ 2-fold less active compared with the 4-propyl parent compound UMW-934, whereas the 3-butyl substituent (analogue **4**, IC_50_ 8.7 µM) was 2-fold more active. These data suggest that the butyl chain length on the 3-position of the phenyl ring is optimal. Subsequently, the tolerance of polar functionality at the 3-position was investigated. The incorporation of a polar group at the 3-position led to a complete loss of activity (analogue **6**, IC_50_ > 50 µM).

**TABLE 1:** Structure–activity relationships of UMW-934 and selected analogues against *Haemonchus contortus*. Inhibitory activity of UMW-934 and selected analogues was assessed against exsheathed third-stage larvae (xL3s) and adult females of *H. contortus*. xL3 motility IC_50_ values and minimum inhibitory concentrations (MIC; concentration achieving 100% inhibition of development to L4) were determined after 168 h of incubation. Adult female motility inhibition was assessed after 24 h exposure at 100 µM and expressed as percentage reduction relative to vehicle controls. Cytotoxicity and mitotoxicity toward HepG2 human hepatoma cells were evaluated after 48 h to determine CC_50_ and MC_50_ values.

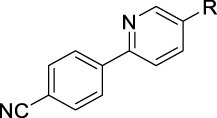						
Compound	Substitution (R)	IC_50_ xL3 (µM)	MIC xL3 (µM)	Adult inhibition (% at 100 µM)	CC_50_ (µM)	MC_50_ (µM)
UMW-934 (1)	n-propyl	18.3	50.0	100 (ii)	–	>40
2	Ethyl	30.0	>50.0	–	–	–
3	Isopropyl	>50.0	>50.0	–	–	–
4	Butyl	8.7	50.0	100 (ii)	–	–
5	Pentyl	27.4	50.0	–	–	–
6	CH_2_NHAc	>50.0	>50.0	0	–	–
7	O-ethyl	16.8	50.0	100 (ii)	>40	–
WEHI-614 (8)	O-propyl	4.9	25.0	–	>40	>40
9	O-butyl	19.3	25.0	100 (ii)	34.0	–
10	O-isobutyl	12.1	50.0	100 (i)	>40	–
11	O-sec-butyl	16.8	50.0	100 (i)	>40	–
12	O-isopentyl	2.7	6.3	87.5	>40	–
13	O-cyclopentyl	26.3	50.0	–	>40	–
14	O-cyclobutylmethyl	3.6	6.3	100 (iii)	>40	–
WEHI-684 (15)	O-cyclohexylmethyl	0.8	3.1	11.1	>40	>40
16	O-oxetan-3-ylmethyl	28.2	50.0	–	>40	–
17	O-phenyl	7.9	12.5	100 (i)	>40	–

Values represent mean measurements from independent experiments as described in Methods. Symbols: “–” indicates not assessed; “>50” indicates no measurable inhibition at the highest concentration tested. Adult inhibition scoring codes: (i) > 70% inhibition at 3 h with 100% inhibition at 12 h; (ii) 40%–70% inhibition at 3 h with 100% inhibition at 24 h; (iii) ∼40% inhibition at 6 h with 100% inhibition at 24 h.

Further diversification of the 3-position on the pyridyl ring with alkoxy and phenoxy substituents yielded multiple derivatives with varying potencies. A 3-ethoxy substituent (analogue **7**, IC_50_ 16.8 µM) was equipotent in comparison to UMW-934. Replacing the ethoxy group with a variety of substituents showed that analogues with n-propyl (**WEHI-614**, IC_50_ 4.9 µM), n-butoxy (analogue **9**, IC_50_ 19.3 µM), isobutyloxy (analogue **10**, IC_50_ 12.1 µM) and sec-butoxy (analogue **11**, IC_50_ 16.8 µM) substituents were equipotent with reference to analogue **7**, whereas cyclopentyloxy (analogue **13**, IC_50_ 26.3 µM) and (oxetane)methoxy (analogue **16**, IC_50_ 28.2 µM) substituents were 2-fold less potent. Interestingly, analogues with 4-isopentyloxy (analogue **12**, IC_50_ 2.7 µM), 4-cyclobutylmethoxy (analogue **14**, IC_50_ 3.6 µM) and 4-cyclohexylmethoxy (**WEHI-684**, IC_50_ 0.8 µM) substituents showed a 4- to 21-fold improvement in activity compared with analogue **7**, whereas the analogue with a 4-phenoxy group (analogue **17**, IC_50_ 7.9 µM) showed a 2-fold increase in activity. Overall, the exploration of the SAR produced analogues achieving low micromolar to sub-micromolar inhibitory concentrations in the xL3 motility assay.

From this optimisation series, two structurally related but distinct derivatives, **WEHI-614** and **WEHI-684**, were prioritised for mechanistic interrogation based on potency, reproducibility and chemical tractability. The availability of multiple active analogues sharing a common scaffold provided an opportunity to assess whether compound-induced proteomic perturbations reflected a scaffold-defined mode-of-action signature rather than analogue-specific effects.

### 2.2 Optimised analogues induce rapid motility impairment across developmental stages

To establish the phenotypic consequences of scaffold optimisation across developmental stages, we evaluated the effects of selected analogues on motility in both exsheathed third-stage larvae and adult females of *H. contortus*. Concentration-dependent inhibition of xL3 motility was confirmed for multiple derivatives, with several analogues achieving marked improvements in *in vitro* potency relative to the parent scaffold (Table 1; Figure 3). In addition to suppressing motility, active compounds impaired larval development to the fourth stage (Table 1) and induced characteristic morphological alterations, including a curved phenotype in affected larvae (Figure 1E).

Importantly, enhanced larval potency translated to activity against adult females. In time-course assays, selected analogues reduced adult female motility progressively over 3–24 h, with several derivatives abolishing movement within 12–24 h at 100 µM (Table 1; Figure 3). These findings confirm that gains in larval potency translate directly to rapid impairment of adult motility, demonstrating preserved stage-translational activity. Cross-species testing revealed variable and generally limited activity against other helminths (Supplementary Table S1), reinforcing *H. contortus* as the principal and most responsive system for this optimisation series.

Notably, motility suppression occurred in the absence of overt mammalian cytotoxicity and mitotoxicity within the tested concentration range (Table 1), supporting a parasite-selective effect rather than general chemical toxicity. The convergence of phenotypic impairment across larval and adult stages suggested that compound exposure disrupts core cellular processes fundamental to nematode movement and physiological integrity.

Given the central role of cytoskeletal organisation and intracellular trafficking in sustaining coordinated motility, we next sought to determine whether scaffold optimisation was associated with reproducible perturbations in structural or regulatory proteins. These compounds (**WEHI-614** and **WEHI-684**) were therefore advanced for proteome-wide thermal profiling and quantitative proteomic analysis.

### 2.3 Proteome integral solubility alteration (PISA) profiling reveals perturbation of cytoskeletal and trafficking proteins

**FIGURE 4:**
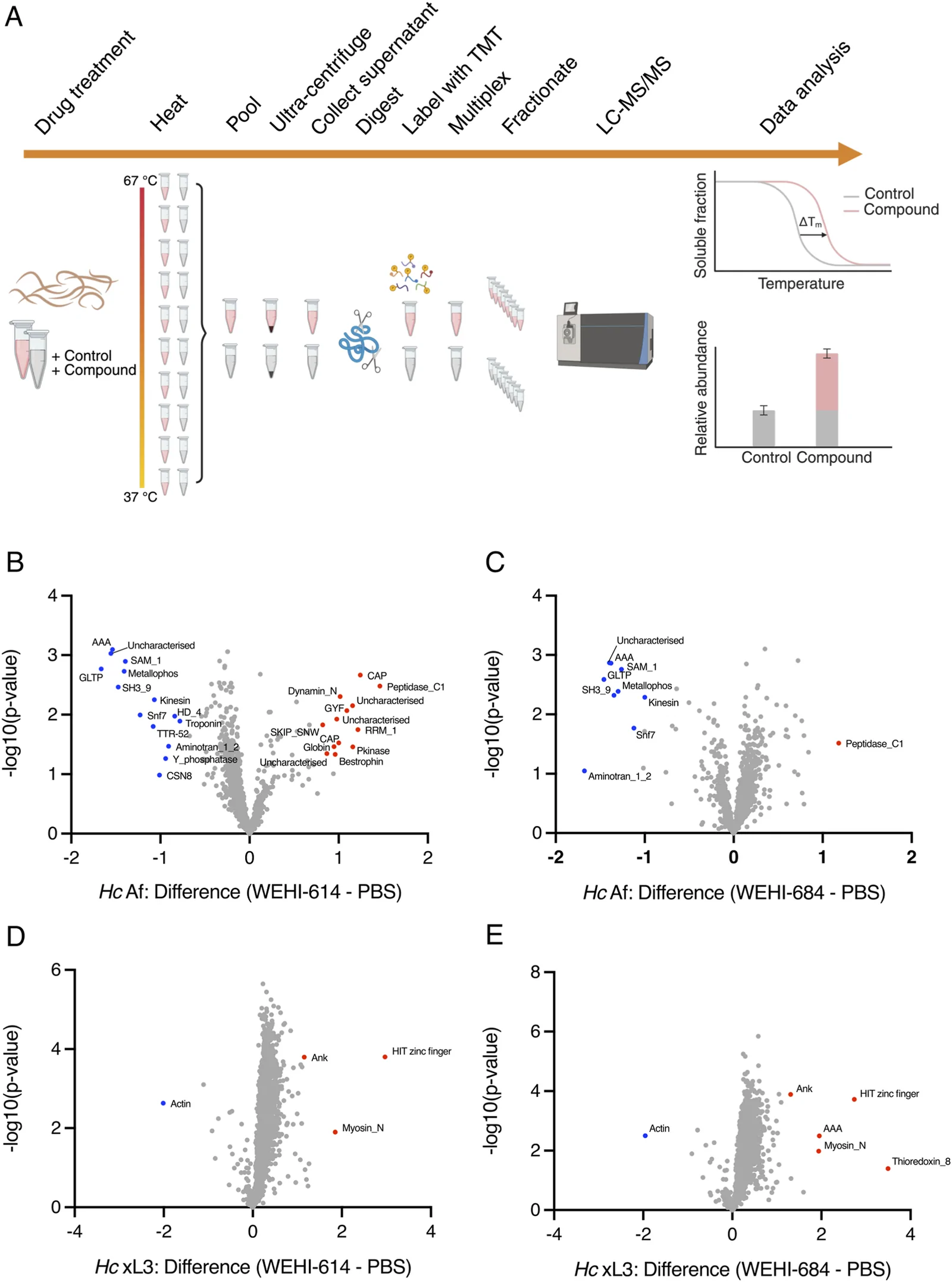
Proteome integral solubility alteration (PISA) profiling identifies reproducible perturbation of cytoskeletal and trafficking proteins in adult and larval stages of *Haemonchus contortus*. **(A)** Schematic overview of the proteome integral solubility alteration (PISA) workflow (modified from Zhang et al., 2022). Soluble protein extracts from adult females or xL3 larvae were incubated with **WEHI-614** or **WEHI-684**, subjected to a defined temperature gradient, and analysed by TMT-based quantitative mass spectrometry to identify proteins exhibiting altered thermal stability relative to PBS controls. **(B)** Volcano plot of thermal stability shifts (ΔTm) in adult females following compound **WEHI-614** exposure. **(C)** Volcano plot of thermal stability shifts (ΔTm) in adult females following compound **WEHI-684** exposure. **(D)** Volcano plot of thermal stability shifts (ΔTm) in xL3 larvae following compound **WEHI-614** exposure. **(E)** Volcano plot of thermal stability shifts (ΔTm) in xL3 larvae following compound **WEHI-684** exposure. Shared and stage-specific solubility alterations are indicated. The vertical axis shows -log10 (p-values), and the horizontal axis shows log2-transformed differences between **WEHI-614** vs. PBS or **WEHI-684** vs. PBS exposures. Putative protein targets ((|ΔTm| ≥0.8, S0 = 0.1 and FDR <0.05; two-sided *t*-tests) are labelled and highlighted in red (increased abundance) and blue (decreased abundance) following compound exposures. The overlap of significantly altered proteins between **WEHI-614** and **WEHI-684** within two developmental stages were underlined.

To define proximal molecular perturbations associated with compound exposure, we performed proteome integral solubility alteration (PISA) profiling on soluble extracts from adult females and xL3 larvae following treatment with **WEHI-614** or **WEHI-684**. This approach integrates protein solubility changes across a defined temperature gradient to identify proteins exhibiting altered thermal stability consistent with compound engagement or proximal perturbation (Figure 4A).

In total, 2,567 and 2,562 soluble proteins were identified and quantified from adult females following separate exposures to **WEHI-614** and **WEHI-684**, respectively (Supplementary Tables S2, S3). *In vitro* exposure to **WEHI-614** resulted in significant solubility alterations in 27 proteins (|ΔTm| ≥0.8, S0 = 0.1 and FDR <0.05), whereas **WEHI-684** affected 10 proteins relative to PBS-treated controls (Figures 4B,C). Nine proteins exhibited altered solubility following treatment with both analogues. Among these, a peptidase C1 family protein (Peptidase_C1; Hcon5G00000008957.1) showed consistent thermal stabilisation across treatments. In contrast, several proteins associated with intracellular trafficking and structural organisation—including kinesin-related motor proteins (Kinesin; Hcon5G00000017279.1), an AAA domain–containing protein (AAA; Hcon5G00000013457.1), components linked to vesicle transport (e.g., Snf7; Hcon5G00000013241.1), and a glycolipid transfer protein (GLTP; Hcon5G00000010893.1), displayed reduced solubility under both conditions. The shared directionality of these solubility shifts across structurally related analogues indicates reproducible perturbation of trafficking and cytoskeletal components rather than analogue-specific effects.

Totals of 2,667 and 2,667 soluble proteins were identified from xL3s following exposure to compounds **WEHI-614** and **WEHI-684**, respectively (Supplementary Tables S4, S5). In xL3 larvae, fewer proteins met statistical thresholds for altered solubility (four for **WEHI-614** and six for **WEHI-684**), yet four proteins were common to both treatments (Figures 4D,E). Notably, actin (Actin; Hcon5G00000016289.1) exhibited decreased solubility following exposure to both analogues. Additional shared alterations included a myosin N-terminal SH3-like domain–containing protein (Myosin_N; Hcon5G00000013622.2) and an ankyrin-domain tyrosine kinase (Ank; Hcon5G00000012261.1), while a HIT zinc finger protein (HIT zinc finger; Hcon5G00000009596.1) displayed consistent thermal stabilisation. The recurrence of actin- and motor-associated proteins across both analogues suggests that structural components of the cytoskeleton are reproducibly perturbed in larval stages.

Across developmental stages, overlapping solubility signatures were observed for two structurally distinct but scaffold-related analogues, supporting a conserved perturbation footprint involving cytoskeletal organisation and intracellular trafficking networks. Because thermal profiling captures changes in protein stability but does not resolve temporal cellular responses, we next asked whether these structural perturbations were accompanied by adaptive changes in protein abundance. To address this, we performed time-resolved quantitative proteomic analysis in adult female worms following compound exposure.

### 2.4 Time-resolved quantitative proteomics identifies proteolytic changes associated with compound exposure

To determine whether the cytoskeletal and trafficking perturbations identified by PISA profiling were accompanied by adaptive changes in protein abundance, we performed time-resolved label-free quantitative proteomic analysis in adult females exposed to **WEHI-614** or **WEHI-684** for four time points (i.e. 0.5 h, 1 h, 3 h and 6 h). Across all time points, 3,320 and 3,332 proteins were identified following exposure to **WEHI-614** and **WEHI-684**, respectively (Supplementary Tables S6, S7).

**FIGURE 5:**
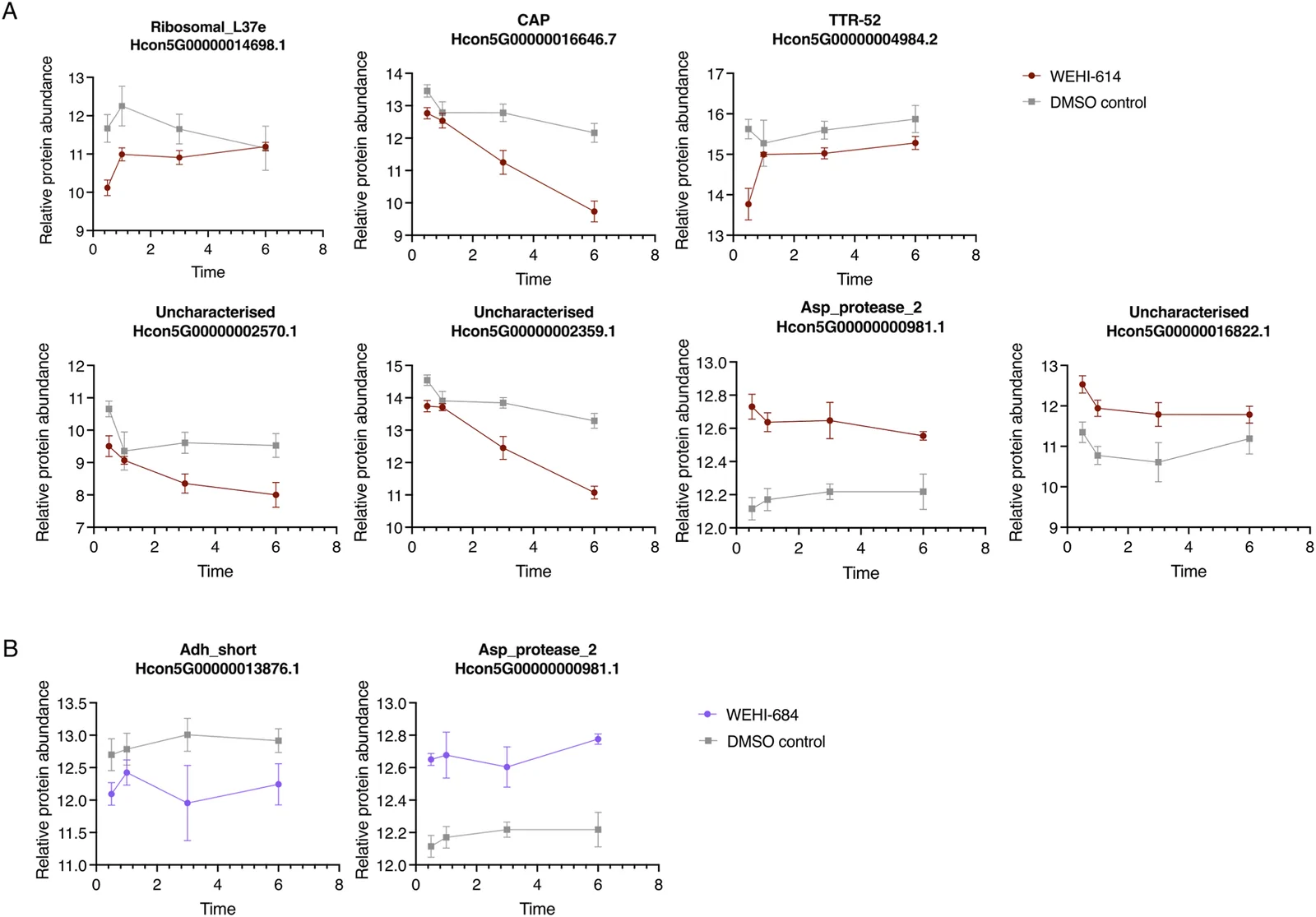
Time-resolved quantitative proteomics identifies limited but reproducible proteolytic changes following compound exposure. Time-resolved quantitative proteomics showing the relative abundance (p < 0.01 and |log_2_ fold change| ≥0.8; two-way ANOVA) of seven and two significantly differentially expressed proteins in adult female *Haemonchus contortus* exposed to **(A) WEHI-614** or **(B) WEHI-684** for 0.5, 1, 3 and 6 h, respectively. Data are presented as mean ± SEM.

Exposure to **WEHI-614** resulted in seven significantly differentially abundant proteins relative to DMSO controls (|log_2_-fold change|: ≥0.8; p < 0.01), five of which decreased and two of which increased over time (Figure 5A). The most pronounced reduction was observed in a cysteine-rich secretory protein belonging to the CAP family (CAP; Hcon5G00000016646.7), which showed a progressive downregulation across the time course. Two additional uncharacterised proteins exhibited similar decreasing trends (encoded by gene Hcon5G00000002359.1 and Hcon5G00000002570.1). In contrast, an aspartyl protease (Asp_protease_2; Hcon5G00000000981.1) displayed a consistent increase in abundance at all time points, although the magnitude of change remained modest (Figure 5A). Following exposure to **WEHI-684**, only two proteins were significantly altered, including Asp_protease_2, which again exhibited sustained upregulation (Figure 5B). The recurrence of this protease response across both analogues suggests a conserved stress response following compound exposure.

Importantly, proteins identified by PISA as exhibiting altered thermal stability were not generally those showing differential abundance over time, indicating a distinction between proximal thermodynamic perturbations and downstream cellular responses. Rather than indicating widespread proteotoxic collapse, the limited and reproducible induction of a proteolytic enzyme suggests activation of a focused stress-adaptive program. Suppression of CAP family proteins, which are associated with secretory and developmental processes in parasitic nematodes (Cantacessi et al., 2009; Mohandas et al., 2015; Liu et al., 2023), further supports physiological impairment following compound exposure.

Taken together, these findings indicate that chemical perturbation of cytoskeletal and trafficking proteins is accompanied by changes in proteolytic pathways. Integration of thermal stability shifts and temporal abundance changes supports a model in which perturbation of structural and trafficking components is associated with secondary modulation of proteolytic processes.

### 2.5 Integrated analysis defines a cytoskeletal and trafficking vulnerability in *Haemonchus contortus*

To identify convergent biological processes underlying compound sensitivity, we integrated thermal stability signatures from PISA with time-resolved abundance changes from quantitative proteomics. Although individual proteins differed between developmental stages and analogues, a consistent functional pattern emerged across datasets.

PISA profiling in both adult females and xL3 larvae revealed reproducible perturbation of proteins associated with cytoskeletal organisation and intracellular trafficking (Figure 4). In larvae, actin and motor-associated proteins exhibited altered solubility following exposure to both analogues, while in adults, kinesin-related proteins, vesicle trafficking components, and ATP-dependent remodelling factors displayed consistent solubility shifts (Figure 4). The recurrence of these structural and trafficking regulators across two chemically distinct but scaffold-related analogues indicates a conserved perturbation footprint rather than analogue-specific effects.

In contrast, time-resolved quantitative proteomics revealed a comparatively restricted set of differentially abundant proteins, dominated by induction of an aspartyl protease and suppression of CAP family secretory proteins (Figure 5). The limited number and modest magnitude of abundance changes argue against widespread proteotoxic collapse and instead suggest involvement of a focused adaptive response. Importantly, the proteolytic induction signature was reproducible across both analogues, consistent with a conserved stress-associated signature.

The integration of thermal stability shifts and temporal abundance changes is consistent with a model in which chemical perturbation of cytoskeletal and trafficking proteins is accompanied by modulation of proteolytic pathways. Given the central role of cytoskeletal architecture in sustaining coordinated motility, intracellular transport, and secretory function, disruption of these interconnected processes would be expected to compromise physiological integrity across developmental stages. The conservation of this signature in both larvae and adults indicates a shared structural–trafficking axis in *H. contortus*. Together, these findings define a cytoskeletal–proteolytic axis revealed by chemical perturbation and associated with secondary proteolytic changes.

## 3 Discussion

The identification of intrinsic biological vulnerabilities in parasitic nematodes is essential for advancing mechanistically distinct anthelmintic strategies in the face of widespread drug resistance (Gasser, 2026). Resistance to multiple drug classes is now established in many livestock systems and increasingly documented in human helminths (Geerts and Gryseels, 2001; Kotze et al., 2014; Charlier et al., 2023; Coffeng et al., 2024), underscoring the limitations of relying exclusively on established pharmacological targets (Shanley et al., 2025). While historically successful anthelmintics have acted on defined molecular entities such as ligand-gated ion channels or β-tubulin (Driscoll et al., 1989; Fleming et al., 1997; Martin et al., 2005; Saunders et al., 2013), these paradigms do not necessarily capture systems-level dependencies that may represent broader axes of chemical sensitivity. In this study, we used an optimised pyridyl scaffold as a chemical probe to interrogate proteome-wide perturbations in *H. contortus*.

Thermal proteome profiling consistently identified altered stability of proteins associated with cytoskeletal organisation and intracellular trafficking. In larvae, actin and motor-associated proteins exhibited reproducible solubility changes following exposure to two structurally related analogues, whereas in adult females, kinesin-related proteins, vesicle trafficking components, and ATP-dependent remodelling factors displayed consistent perturbations. Cytoskeletal architecture underpins coordinated locomotion in nematodes, where actin–myosin dynamics and motor proteins sustain contractility and force transmission (Gieseler et al., 2017). Beyond mechanical function, the cytoskeleton is structurally and functionally coupled to intracellular trafficking systems, including vesicle transport and membrane remodelling (Yang et al., 2020). ATP-dependent remodelling complexes and trafficking adaptors regulate organelle positioning, endosomal sorting and membrane turnover (e.g., Stoten and Carlton, 2018). The recurrence of perturbations in these interconnected regulators across developmental stages indicates that chemical exposure reproducibly affects structural and trafficking networks central to parasite physiology. Importantly, this perturbation profile differs conceptually from classical microtubule-targeting agents that directly disrupt polymer assembly (Wordeman and Vicente, 2021). Instead of collapsing a single structural element, the present data indicate solubility shifts for multiple proteins involved in cytoskeletal organisation and transport. Such distributed perturbation may reflect interference with interconnected components that integrate structural dynamics and trafficking flux. Because thermal profiling detects changes in protein stability consistent with compound-associated or proximal perturbation (Jafari et al., 2014), these findings suggest that cytoskeletal and trafficking proteins represent key loci of chemical sensitivity.

In contrast to the broad structural perturbation signature observed by PISA, time-resolved quantitative proteomics revealed a comparatively restricted set of differentially abundant proteins. Induction of an aspartyl protease was reproducibly observed across both analogues, while suppression of CAP family secretory proteins and selected additional proteins occurred in a compound-dependent manner. Aspartyl proteases contribute to nutrient processing, host interaction and stress adaptation in parasitic nematodes (Lin and Siddique, 2024), and CAP family proteins are associated with secretory and developmental functions (Cantacessi et al., 2009; Mohandas et al., 2015; Liu et al., 2023). The limited number and moderate magnitude of abundance changes argue against global proteotoxic collapse. Instead, the reproducible induction of a proteolytic enzyme supports activation of a focused stress-associated response. Moreover, the separation between proximal stability perturbations and downstream abundance modulation indicates that thermal stability shifts are not generally mirrored by changes in protein abundance over time. Nevertheless, the restricted abundance changes observed within the early profiling window are not inconsistent with the marked motility impairment recorded in adult females, as rapid functional consequences may additionally arise through mechanisms not captured by total protein abundance measurements; extending the time course in future proteomic studies would provide greater resolution of the downstream response.

Cytoskeletal and trafficking systems are tightly integrated in metazoan cells, where structural scaffolds coordinate transport, signalling and membrane turnover (Fletcher and Mullins, 2010). In parasitic helminths, these systems support locomotion, tissue invasion, secretion of effector molecules and nutrient acquisition (Mekonnen et al., 2018; Drurey and Maizels, 2021). Destabilisation of this integrated network would likely compromise motility and disrupt physiological homeostasis.

The conservation of structural perturbation signatures across larval and adult stages indicates that this dependency is not stage-restricted; notably, this conservation reflects pathway-level rather than molecular identity-level similarity, and represents a shared sensitivity intrinsic to *H. contortus* biology. Such stage-spanning sensitivity is notable, as developmental transitions in nematodes typically involve extensive remodelling of regulatory and structural networks (Ma et al., 2018; Wang et al., 2019; Wang et al., 2025). The persistence of cytoskeletal–trafficking perturbation signatures across these contexts supports the existence of a conserved structural–trafficking axis fundamental to parasite physiology.

The implications of a cytoskeletal–trafficking vulnerability extend to resistance considerations. Single-enzyme targets are vulnerable to resistance through point mutations or target modification (Kotze et al., 2014; Shanley et al., 2025). In contrast, perturbation of interconnected structural and trafficking networks may require coordinated compensatory adaptations, potentially reducing the likelihood of rapid resistance emergence. Although this hypothesis requires direct experimental testing, it raises the possibility that network-level vulnerabilities could represent more resilient intervention points than single-protein targets (Hopkins, 2008; Lanusse et al., 2018).

Several potential limitations merit consideration. Thermal profiling identifies proteins exhibiting altered stability but does not establish direct molecular binding (Mateus et al., 2020; Figueroa-Navedo and Ivanov, 2024). Stability shifts may reflect indirect or proximal effects within multiprotein complexes. Similarly, quantitative proteomics reveals abundance changes that may represent adaptive responses rather than primary lesions (Franken et al., 2015). The present data therefore define a convergent network-level perturbation signature rather than identifying a definitive molecular target. Future biochemical, genetic and cellular studies will be required to resolve direct compound–protein interactions and establish causal links between proteomic perturbations and motility impairment. In addition, the limited activity observed against other helminth species suggests species-selective sensitivity, potentially reflecting differences in compound uptake, cuticle permeability, metabolism, or divergence in the sequence or structure of proteins associated with the perturbed pathways. This observation reinforces *H. contortus* as the principal system for further optimisation and mechanistic investigation of this scaffold.

In conclusion, integration of thermal proteome profiling and time-resolved quantitative proteomics indicates that chemical perturbation exposes a cytoskeletal and trafficking vulnerability in *H. contortus*, accompanied by reproducible modulation of proteolytic pathways. Rather than implicating a single dominant molecular target, these findings support a model in which disruption of structural and intracellular transport processes contributes to rapid impairment of motility and larval development. Defining such systems-level dependencies provides a framework for understanding chemical susceptibility in parasitic nematodes and may inform the rational development of mechanistically distinct anthelmintic strategies.

## 4 Materials and methods

### 4.1 Parasites

*Haemonchus contortus* (Haecon-5 strain) was maintained in experimentally infected sheep using established protocol (Taki et al., 2021). Helminth-free Merino sheep (6 months of age; male) were orally inoculated with 7,000 L3s. Four weeks post-infection, faecal samples containing eggs were collected daily. L3s were generated by incubating faecal material at 27 °C and >90% relative humidity for 7 days, recovered, sieved (20 µm mesh), and stored in water at 11 °C for up to 6 months. Immediately prior to assays, L3s were exsheathed and sterilised by incubation in 0.15% bleach at 38 °C for 20 min, washed five times in sterile saline, and resuspended in LB* (lysogeny broth supplemented with penicillin, streptomycin, and amphotericin B). Adult females were collected from the abomasum of infected sheep at necropsy (4 weeks post-infection), washed extensively in phenol red–free RPMI 1640 supplemented with antimicrobials (RPMI*), and used immediately.

For comparative cross-species assessment, additional helminths were maintained using established protocols (Lombardo et al., 2019; Buchter et al., 2021; Keiser and Häberli, 2021). *Ancylostoma ceylanicum* and *Necator americanus* were maintained in Syrian golden hamsters, *Heligmosomoides polygyrus* and *Schistosoma mansoni* in NMRI mice, *Trichuris muris* in C57BL/6NRj mice, and *Strongyloides ratti* in Wistar rats. Infective nematode stages were produced from faeces (8–10 days at 24 °C and >90% humidity) and larvae recovered using the Baermann technique (*S. ratti*) or cultivation on agar plates. *Sh*. *Mansoni* newly transformed schistosomula (NTS) were prepared by mechanical transformation of cercariae shed from infected intermediate host *Biomphalaria glabrata* snails. Larvae were maintained in species-appropriate media (HBSS, RPMI 1640, M199 or PBS) supplemented with antimicrobials. Adult worms were collected from host intestines at standard time points post-infection and maintained in supplemented culture media at 37 °C in 5% CO_2_ prior to use.

### 4.2 Compound preparation

UMW-934 and analogues were dissolved in DMSO and diluted into assay media. Final DMSO concentration was 0.25% unless otherwise stated. Synthetic procedures and compound characterisation are described in the Supplementary Methods.

### 4.3 Motility and viability assays

For *H. contortus*, exsheathed L3s (300 per well) were dispensed into 96-well plates in 100 µL LB* and exposed to compounds as 9-point, 2-fold serial dilutions (50–0.2 µM) following established protocols (Taki et al., 2021; Taki et al., 2022). Plates were incubated at 38 °C, 10% CO_2_ and >90% relative humidity, and motility and development to L4 were assessed after 168 h. Concentration–response data were log_10_-transformed and fitted using a variable-slope four-parameter model in GraphPad Prism (v11.0.0).

Adult female *H. contortus* (3 worms per well) were incubated in 24-well plates containing 500 µL RPMI* with compound (100 µM) at 38 °C and 10% CO_2_ for up to 24 h. Motility was recorded (30 s) and scored on a 0–3 scale (3, normal; 0, no movement) and normalised to vehicle controls (Taki et al., 2021).

For comparative cross-species assessment, L3s of *A. ceylanicum*, *N. americanus*, *He. Polygyrus* and *S. ratti* (30–40 larvae per well), adults of *A. ceylanicum*, *S. ratti* and *T. muris* (three to six worms per well) and NTS of *Sh. Mansoni* (50 NTS per well) were incubated with compounds at 10 µM (50 µM for *He. polygyrus*) under species-appropriate conditions (22 °C–37 °C, ±5% CO_2_, high humidity). Larval death and adult viability were assessed after 72 h based on motility following thermal stimulation (∼80 °C) using established scoring systems (Keiser et al., 2016; Keiser and Häberli, 2021). Experiments were performed in replicate wells.

### 4.4 Cytotoxicity and mitotoxicity assays

HepG2 human hepatoma cells were maintained in DMEM supplemented with 10% heat-inactivated FBS and antimicrobials at 37 °C in 5% CO_2_. HepG2 cells were seeded at 5.5 × 10^4^ cells per well in 96-well plates and allowed to adhere for 16 h. Compounds were tested as 7-point, 2-fold serial dilutions (50 μM–0.78 µM) for 48 h. For mitotoxicity, cells were serum-starved for 4 h prior to treatment and maintained in galactose-based medium (Swiss and Will, 2011; Kamalian et al., 2015). Cell viability was assessed by crystal violet staining (absorbance 595 nm) (Śliwka et al., 2016). CC_50_ and MC_50_ values were determined by four-parameter curve fitting in GraphPad Prism.

### 4.5 Proteome integral solubility alteration (PISA) profiling

Protein extracts were prepared from 30,000 xL3s or 50 adult females. Worms were pelleted (2,000 × g, 5 min), snap-frozen, and resuspended in 400 µL PBS (pH 7.0) containing 0.5% NP-40 and protease inhibitor cocktail. Samples were sonicated on ice using a BioRuptor (20 kHz; eight cycles of 20 s on/20 s off) and clarified by centrifugation (20,000 × g, 20 min, 4 °C). Protein concentration was determined by BCA assay and adjusted to 2 μg/μL.

Somatic proteins (60 µL) were incubated with compound at approximately 10 × LC_50_ (**WEHI-614**: 49 μM; **WEHI-684**: 8.5 µM) or PBS control for 30 min at 23 °C (three biological replicates). Samples were aliquoted into 10 PCR tubes and heated for 3 min at 37, 41, 44, 47, 50, 53, 56, 59, 63 °C and 67 °C, respectively. Aliquots were recombined, centrifuged (20,000 × g, 20 min, 4 °C), and soluble fractions collected.

Proteins were reduced (10 mM TCEP), alkylated (55 mM iodoacetamide), and digested with trypsin (16 h, 37 °C). Peptides were desalted, labelled with TMT10pLex reagents (TMT10-131 reference channel) using an established protocol (Zecha et al., 2019), fractionated into 8 fractions, and analysed by LC–MS/MS on an Orbitrap Ascend coupled to an Ultimate 3000 RSLC system.

Data were processed using MaxQuant (v2.7.5.0) against the Haecon-5 predicted proteome with protein and PSM FDR <1%. Differential solubility between compound and control samples was assessed using two-sided t-tests in Perseus (v.2.1.5.0) (Tyanova et al., 2016), retaining proteins detected in all biological replicates. Volcano plot analysis was performed with an S0 parameter of 0.1, combining fold-change and t-test p-value to rank significance. Proteins with absolute thermal stability shifts (ΔTm) ≥ 0.8 and FDR <0.05 were considered as significant.

### 4.6 Time-resolved label-free quantitative proteomics

Adult females (*n* = 5 per group) were exposed to WEHI-614, WEHI-684 or DMSO for 0.5, 1, 3 and 6 h (four biological replicates per time point). Proteins were extracted in 8 M urea in 100 mM TEAB (pH 8.5), reduced, alkylated, and digested with trypsin (18 h, 37 °C) as described previously (Wang et al., 2025).

Data-independent acquisition (DIA) LC–MS/MS was performed on an Orbitrap Astral using 2 Da isolation windows (13.7 Da with 1 Da overlap). Data were analysed using Spectronaut (v19.4.241104.62635) with protein and PSM FDR set to 1%. Quantitative data were exported for statistical analyses using Perseus (v.2.1.5.0) (Tyanova et al., 2016). Briefly, protein intensities were log_2_-transformed. Proteins with more than two missing values per group were excluded; remaining missing values were imputed (width 0.3; downshift 1.8). Treatment effects across time were tested using two-way ANOVA. Proteins were considered differentially abundant if p < 0.01 and |log_2_ fold change| ≥0.8 (corresponding to ∼1.74-fold change in relative protein abundance).

## Data Availability

The PISA proteomic data and the time-resolved LFQ proteomics data have been deposited in the PRIDE repository and are publicly available under accession numbers PXD075263 and PXD075211, respectively.

## Supplementary material

SUPPLEMENTARY METHODS Chemistry experimental.

SUPPLEMENTARY TABLE S1 Cross-species *in vitro* activity of UMW-934 and selected analogues.

SUPPLEMENTARY TABLE S2 Results from proteome integral solubility alteration (PISA) profiling of adult females of *Haemonchus contortus* following exposure to WEHI-614.

SUPPLEMENTARY TABLE S3 Results from proteome integral solubility alteration (PISA) profiling of adult females of *Haemonchus contortus* following exposure to WEHI-684.

SUPPLEMENTARY TABLE S4 Results from proteome integral solubility alteration (PISA) profiling of xL3 larvae of *Haemonchus contortus* following exposure to WEHI-614.

SUPPLEMENTARY TABLE S5 Results from proteome integral solubility alteration (PISA) profiling of xL3 larvae of *Haemonchus contortus* following exposure to WEHI-684.

SUPPLEMENTARY TABLE S6 Results from time-resolved label-free quantitative proteomic analysis of adult female *Haemonchus contortus* following exposure to WEHI-614.

SUPPLEMENTARY TABLE S7 Results from time-resolved label-free quantitative proteomic analysis of adult female *Haemonchus contortus* following exposure to WEHI-684.

## References

[B1] Batth T. S. Locard-Paulet M. Doncheva N. T. Lopez Mendez B. Jensen L. J. Olsen J. V. (2024). Streamlined analysis of drug targets by proteome integral solubility alteration indicates organ-specific engagement. Nat. Commun. 15, 8923. 10.1038/s41467-024-53240-2 39414818 PMC11484808

[B2] Baxi A. B. Nemes P. Moody S. A. (2023). Time-resolved quantitative proteomic analysis of the developing *Xenopus* otic vesicle reveals putative congenital hearing loss candidates. iScience 26, 107665. 10.1016/j.isci.2023.107665 37670778 PMC10475516

[B3] Buchter V. Hofmann D. Häberli C. Keiser J. (2021). Characterization of moxidectin against *Strongyloides ratti: in vitro* and *in vivo* activity and pharmacokinetics in the rat model. ACS Infect. Dis. 7, 1069–1076. 10.1021/acsinfecdis.0c00435 32991142

[B4] Burns A. R. Luciani G. M. Musso G. Bagg R. Yeo M. Zhang Y. (2015). *Caenorhabditis elegans* is a useful model for anthelmintic discovery. Nat. Commun. 6, 7485. 10.1038/ncomms8485 26108372 PMC4491176

[B5] Cantacessi C. Campbell B. E. Visser A. Geldhof P. Nolan M. J. Nisbet A. J. (2009). A portrait of the “SCP/TAPS” proteins of eukaryotes — developing a framework for fundamental research and biotechnological outcomes. Biotechnol. Adv. 27, 376–388. 10.1016/j.biotechadv.2009.02.005 19239923

[B6] Charlier J. Bartley D. J. Sotiraki S. Martinez-Valladares M. Claerebout E. von Samson-Himmelstjerna G. (2022). Anthelmintic resistance in ruminants: challenges and solutions. Adv. Parasitol. 115, 171–227. 10.1016/bs.apar.2022.01.002 35249662

[B7] Charlier J. Hoste H. Sotiraki S. (2023). COMBAR – combatting anthelmintic resistance in ruminants. Parasite 30, E1. 10.1051/parasite/2023001 36762940 PMC9912929

[B8] Coffeng L. E. Stolk W. A. de Vlas S. J. (2024). Predicting the risk and speed of drug resistance emerging in soil-transmitted helminths during preventive chemotherapy. Nat. Commun. 15, 1099. 10.1038/s41467-024-46166-1 38321011 PMC10847116

[B9] Cortés A. Sánchez-López C. M. Gónzalez-Arce A. Bernal D. Marcilla A. (2025). Helminth extracellular vesicles: roles in and beyond host-parasite communication. Adv. Parasitol. 128, 1–34. 10.1016/bs.apar.2025.01.001 40947160

[B10] Driscoll M. Dean E. Reilly E. Bergholz E. Chalfie M. (1989). Genetic and molecular analysis of a *Caenorhabditis elegans* beta-tubulin that conveys benzimidazole sensitivity. J. Cell. Biol. 109, 2993–3003. 10.1083/jcb.109.6.2993 2592410 PMC2115974

[B11] Drurey C. Maizels R. M. (2021). Helminth extracellular vesicles: interactions with the host immune system. Mol. Immunol. 137, 124–133. 10.1016/j.molimm.2021.05.003 34246032 PMC8636279

[B12] Figueroa-Navedo A. M. Ivanov A. R. (2024). Experimental and data analysis advances in thermal proteome profiling. Cell. Rep. Methods 4, 100717. 10.1016/j.crmeth.2024.100717 38412830 PMC10921035

[B13] Fleming J. T. Squire M. D. Barnes T. M. Tornoe C. Matsuda K. Ahnn J. (1997). *Caenorhabditis elegans* levamisole resistance genes lev-1, unc-29, and unc-38 encode functional nicotinic acetylcholine receptor subunits. J. Neurosci. 17, 5843–5857. 10.1523/JNEUROSCI.17-15-05843.1997 9221782 PMC6573193

[B14] Fletcher D. A. Mullins R. D. (2010). Cell mechanics and the cytoskeleton. Nature 463, 485–492. 10.1038/nature08908 20110992 PMC2851742

[B15] Franken H. Mathieson T. Childs D. Sweetman G. M. A. Werner T. Tögel I. (2015). Thermal proteome profiling for unbiased identification of direct and indirect drug targets using multiplexed quantitative mass spectrometry. Nat. Protoc. 10, 1567–1593. 10.1038/nprot.2015.101 26379230

[B16] Gaetani M. Sabatier P. Saei A. A. Beusch C. M. Yang Z. Lundström S. L. (2019). Proteome integral solubility alteration: a high-throughput proteomics assay for target deconvolution. J. Proteome Res. 18, 4027–4037. 10.1021/acs.jproteome.9b00592 31545609

[B69] Gasser R. B. (2026). Biotechnology advances and the parasitology paradigm: from genomes to multi-omics and translation. Biotechnol. Adv. 89, 108813. 10.1016/j.biotechadv.2026.108813 41628813

[B17] Geary T. G. Thompson D. P. (2001). *Caenorhabditis elegans:* how good a model for veterinary parasites? Vet. Parasitol. 101, 371–386. 10.1016/S0304-4017(01)00530-8 11707307

[B18] Geerts S. Gryseels B. (2001). Anthelmintic resistance in human helminths: a review. Trop. Med. Int. Health 6, 915–921. 10.1046/j.1365-3156.2001.00890.x 11703846

[B19] Gieseler K. Qadota H. Benian G. M. (2017). Development, structure, and maintenance of *C. elegans* body wall muscle. WormBook 2017, 1–59. 10.1895/wormbook.1.81.2 27555356 PMC5410635

[B20] Hahnel S. R. Dilks C. M. Heisler I. Andersen E. C. Kulke D. (2020). *Caenorhabditis elegans* in anthelmintic research – old model, new perspectives. Int. J. Parasitol. Drugs Drug Resist. 14, 237–248. 10.1016/j.ijpddr.2020.09.005 33249235 PMC7704361

[B21] Herath H. M. P. D. Taki A. C. Rostami A. Jabbar A. Keiser J. Geary T. G. (2022). Whole-organism phenotypic screening methods used in early-phase anthelmintic drug discovery. Biotechnol. Adv. 40, 107937. 10.1016/j.biotechadv.2022.107937 35271946

[B22] Holden-Dye L. Walker R. J. (2014). “Anthelmintic drugs and nematicides: studies in Caenorhabditis elegans,” in *WormBook.* Pasadena, CA: The C. elegans Research Community, 1–29. 10.1895/wormbook.1.143.2 PMC5402214 25517625

[B23] Hopkins A. L. (2008). Network pharmacology: the next paradigm in drug discovery. Nat. Chem. Biol. 4, 682–690. 10.1038/nchembio.118 18936753

[B24] Jafari R. Almqvist H. Axelsson H. Ignatushchenko M. Lundbäck T. Nordlund P. (2014). The cellular thermal shift assay for evaluating drug target interactions in cells. Nat. Protoc. 9, 2100–2122. 10.1038/nprot.2014.138 25101824

[B25] Jiao Y. Preston S. Hofmann A. Taki A. Baell J. Chang B. C. H. (2020). A perspective on the discovery of selected compounds with anthelmintic activity against the barber’s pole worm—where to from here? Adv. Parasitol. 108, 1–45. 10.1016/bs.apar.2020.07.001 32291083

[B26] Kamalian L. Chadwick A. E. Bayliss M. French N. S. Monshouwer M. Snoeys J. (2015). The utility of HepG2 cells to identify direct mitochondrial dysfunction in the absence of cell death. Toxicol. In Vitro 29, 732–740. 10.1016/j.tiv.2015.02.011 25746382

[B27] Kaminsky R. Mäser P. (2025). Global impact of parasitic infections and the importance of parasite control. Front. Parasitol. 4, 1546195. 10.3389/fpara.2025.1546195 40129690 PMC11931396

[B28] Keiser J. Häberli C. (2021). Evaluation of commercially available anthelmintics in laboratory models of human intestinal nematode infections. ACS Infect. Dis. 7, 1177–1185. 10.1021/acsinfecdis.1c00079 33410658

[B29] Keiser J. Panic G. Adelfio R. Cowan N. Vargas M. Scandale I. (2016). Evaluation of an FDA approved library against laboratory models of human intestinal nematode infections. Parasit. Vectors 9, 376. 10.1186/s13071-016-1651-9 27363703 PMC4929775

[B30] Kotze A. C. Hunt P. W. Skuce P. von Samson-Himmelstjerna G. Martin R. J. Sager H. (2014). Recent advances in candidate-gene and whole-genome approaches to the discovery of anthelmintic resistance markers and drug/receptor interactions. Int. J. Parasitol. Drugs Drug Resist. 4, 164–184. 10.1016/j.ijpddr.2014.05.001 25516826 PMC4266812

[B31] Lanusse C. Canton C. Virkel G. Alvarez L. Costa-Junior L. Lifschitz A. (2018). Strategies to optimize the efficacy of anthelmintic drugs in ruminants. Trends Parasitol. 34, 664–682. 10.1016/j.pt.2018.04.003 29960843

[B32] Lin C. J. Siddique S. (2024). Parasitic nematodes: dietary habits and their implications. Trends Parasitol. 40, 230–240. 10.1016/j.pt.2023.11.002 38262837

[B33] Liu H. Tao Z. Wang Y. Liu X. Wang C. Liu L. (2023). A member of the CAP protein superfamily, *Hc*-CAP-15, is important for the parasitic-stage development of *Haemonchus contortus*. Parasit. Vectors 16, 290. 10.1186/s13071-023-05907-w 37592312 PMC10433639

[B34] Lok J. B. (2016). Signaling in parasitic nematodes: physicochemical communication between host and parasite and endogenous molecular transduction pathways governing worm development and survival. Curr. Clin. Microbiol. Rep. 3, 186–197. 10.1007/s40588-016-0041-2 28781934 PMC5543980

[B35] Lombardo F. C. Pasche V. Panic G. Endriss Y. Keiser J. (2019). Life cycle maintenance and drug-sensitivity assays for early drug discovery in *Schistosoma mansoni*. Nat. Protoc. 14, 461–481. 10.1038/s41596-018-0101-y 30610241

[B36] Ma G. Wang T. Korhonen P. K. Ang C.-S. Williamson N. A. Young N. D. (2018). Molecular alterations during larval development of *Haemonchus contortus in vitro* are under tight post-transcriptional control. Int. J. Parasitol. 48, 763–772. 10.1016/j.ijpara.2018.03.008 29792880

[B37] Ma G. Wang T. Korhonen P. K. Hofmann A. Sternberg P. W. Young N. D. (2020). Elucidating the molecular and developmental biology of parasitic nematodes: moving to a multiomics paradigm. Adv. Parasitol. 108, 175–229. 10.1016/bs.apar.2019.12.005 32291085

[B38] Maity R. Zhang X. Liberati F. R. Scribani Rossi C. Cutruzzola F. Rinaldo S. (2024). Merging multi-omics with proteome integral solubility alteration unveils antibiotic mode of action. eLife 13, e98745. 10.7554/eLife.96343 PMC11434622 39329363

[B70] Marhöfer R. J. Noack S. Selzer P. M. (2025). Antiparasitics discovery: from genotype to phenotype to compounds. Trends Parasitol. 41, 431–440. 10.1016/j.pt.2025.04.007 40345885

[B39] Martin R. J. Verma S. Levandoski M. Clark C. L. Qian H. Stewart M. (2005). Drug resistance and neurotransmitter receptors of nematodes: recent studies on levamisole. Parasitology 131, S71–S84. 10.1017/S0031182005007227 16569294

[B40] Mateus A. Kurzawa N. Becher I. Sridharan S. Helm D. Stein F. (2020). Thermal proteome profiling for interrogating protein interactions. Mol. Syst. Biol. 16, e9232. 10.15252/msb.20199232 32133759 PMC7057112

[B41] Mekonnen G. G. Pearson M. Loukas A. Sotillo J. (2018). Extracellular vesicles from parasitic helminths and their potential utility as vaccines. Expert Rev. Vaccines 17, 197–205. 10.1080/14760584.2018.1425616 29353519

[B42] Moffat J. G. Vincent F. Lee J. A. Eder J. Prunotto M. (2017). Opportunities and challenges in phenotypic drug discovery. Nat. Rev. Drug Discov. 16, 531–543. 10.1038/nrd.2017.111 28685762

[B43] Mohandas N. Young N. D. Jabbar A. Korhonen P. K. Koehler A. V. Amani P. (2015). The barber’s pole worm CAP protein superfamily. Biotechnol. Adv. 33, 1744–1754. 10.1016/j.biotechadv.2015.07.002 26239368

[B44] Monastyrska I. Rieter E. Klionsky D. J. Reggiori F. (2009). Multiple roles of the cytoskeleton in autophagy. Biol. Rev. 84, 431–448. 10.1111/j.1469-185X.2009.00077.x 19659885 PMC2831541

[B45] Moreno Y. Geary T. G. Tritten L. (2021). When secretomes meet anthelmintics. Trends Parasitol. 37, 468–475. 10.1016/j.pt.2021.01.005 33563557

[B46] Nixon S. A. Welz C. Woods D. J. Costa-Junior L. Zamanian M. Martin R.J. (2020). Where are all the anthelmintics? Int. J. Parasitol. Drugs Drug Resist. 14, 8–16. 10.1016/j.ijpddr.2020.07.001 32814269 PMC7452592

[B47] Partridge F. A. Forman R. Bataille C. J. R. Wynne G. M. Nick M. Russell A. J. (2020). Anthelmintic drug discovery. Beilstein J. Org. Chem. 16, 1203–1224. 10.3762/bjoc.16.103 32550933 PMC7277699

[B48] Pasquer Q. T. L. Tsakoumagkos I. A. Hoogendoorn S. (2020). From phenotypic hit to chemical probe. Molecules 25, 5708. 10.3390/molecules25235708 33287212 PMC7730769

[B49] Perrin J. Werner T. Kurzawa N. Rutkowska A. Childs D. D. Kalxdorf M. (2020). Identifying drug targets in tissues and whole blood. Nat. Biotechnol. 38, 303–308. 10.1038/s41587-019-0386-7 31959954

[B50] Roy P. J. (2025). Drug screens using the nematode *Caenorhabditis elegans*. Genetics 231, iyaf141. 10.1093/genetics/iyaf141 40796140 PMC12406009

[B51] Saunders G. I. Wasmuth J. D. Beech R. Laing R. Hunt M. Naghra H. (2013). Characterization of *Haemonchus contortus* β-tubulin gene family. Int. J. Parasitol. 43, 465–475. 10.1016/j.ijpara.2013.02.001 23416426

[B52] Selzer P. M. Epe C. (2021). Antiparasitics in animal health. Trends Parasitol. 37, 77–89. 10.1016/j.pt.2020.10.001 33039282

[B53] Shanley H. T. Wang T. Taki A. C. Byrne J. J. Chang B. C. H. Sleebs B. E. (2025). Advances in anthelmintic target identification. Int. J. Mol. Sci. 26, 3738. 10.3390/ijms26053738 40332360 PMC12028019

[B54] Śliwka L. Wiktorska K. Suchocki P. Milczarek M. Mielczarek S. Lubelska K. (2016). Comparison of MTT and CVS assays. PLoS One 11, e0155772. 10.1371/journal.pone.0155772 27196402 PMC4873276

[B55] Stoten C. L. Carlton J. G. (2018). ESCRT-dependent control of membrane remodelling. Semin. Cell. Dev. Biol. 74, 50–65. 10.1016/j.semcdb.2017.08.020 28843980 PMC6015221

[B56] Swiss R. Will Y. (2011). Assessment of mitochondrial toxicity in HepG2 cells cultured in high-glucose- or galactose-containing media. Curr. Protoc. Toxicol. 49, 2.20.1–2.20.14. 10.1002/0471140856.tx0220s49 21818751

[B57] Taki A. C. Byrne J. J. Wang T. Sleebs B. E. Nguyen N. Hall R. S. (2021). High-throughput phenotypic assay for *Haemonchus contortus*. Pharmaceuticals 14, 616. 10.3390/ph14070616 34206910 PMC8308562

[B58] Taki A. C. Wang T. Nguyen N. N. Ang C.-S. Leeming M. G. Nie S. (2022). Thermal proteome profiling reveals *Haemonchus* orphan protein HCO_011565. Front. Pharmacol. 13, 1014804. 10.3389/fphar.2022.1014804 36313370 PMC9616048

[B59] Tyanova S. Temu T. Sinitcyn P. Carlson A. Hein M. Y. Geiger T. (2016). The Perseus computational platform. Nat. Methods 13, 731–740. 10.1038/nmeth.3901 27348712

[B60] Wang T. Ma G. Ang C.-S. Korhonen P. K. Xu R. Nie S. (2019). Somatic proteome of *Haemonchus contortus*. Int. J. Parasitol. 49, 311–320. 10.1016/j.ijpara.2018.11.003 30771357

[B61] Wang T. Zheng Y. Young N. D. Ang C.-S. Gasser R. B. (2025). Developmental somatic proteome atlas of *Haemonchus contortus*. Parasit. Vectors 18, 379. 10.1186/s13071-025-07022-8 40993749 PMC12462265

[B62] Wordeman L. Vicente J. J. (2021). Microtubule targeting agents in disease. Cancers 13, 5650. 10.3390/cancers13225650 34830812 PMC8616087

[B63] Yang Z. Mattingly B. C. Hall D. H. Ackley B. D. Buechner M. (2020). Terminal web and vesicle trafficking proteins. J. Cell. Biol. 219, e202003152. 10.1083/jcb.202003152 32860501 PMC7594493

[B64] Yang N. Matthew M. A. Yao C. (2023). Roles of cysteine proteases in parasites. Microorganisms 11, 1397. 10.3390/microorganisms11061397 37374899 PMC10300726

[B65] Zajíčková M. Nguyen L. T. Skálová L. Stuchlíková L. R. Matoušková P. (2019). Anthelmintics in the future. Drug Discov. Today 24, 1229–1238. 10.1016/j.drudis.2019.02.001 31883953

[B66] Zecha J. Satpathy S. Kanashova T. Avanessian S. C. Kane M. H. Clauser K. R. (2019). TMT labeling for the masses. Mol. Cell. Proteomics 18, 1468–1478. 10.1074/mcp.TIR119.001608 30967486 PMC6601210

[B67] Zhang X. Lytovchenko O. Lundström S. Zubarev R. Gaetani M. (2022). PISA assay in mammalian cells. Bio-Protoc. 12, e4560. 10.21769/BioProtoc.4560 36532690 PMC9724010

[B68] Zheng Y. Young N. D. Wang T. Chang B. C. H. Song J. Gasser R. B. (2025). Systems biology of *Haemonchus contortus*. Biotechnol. Adv. 81, 108567. 10.1016/j.biotechadv.2025.108567 40127743

