# Supplementary material for: Chemical perturbation reveals a cytoskeletal–trafficking vulnerability in Haemonchus contortus
Source: Front Pharmacol. 2026 Aug 14;17:1866526. doi: 10.3389/fphar.2026.1866526 (PMC13522907; doi:10.3389/fphar.2026.1866526)
Supplement: Supplementary file 1 [file DataSheet1.pdf]

SUPPLEMENTAL INFORMATION

Chemical perturbation reveals a cytoskeletal–trafficking vulnerability in *Haemonchus contortus*

Aya C. Taki[1†], Nghi Nguyen[2†], Tao Wang[1†], Joseph J. Byrne[1], Anson V. Koehler[1], Harrison T. Shanley[1,2], Ching-Seng Ang[3], Jennifer Keiser[4], Cécile Häberli[4], Brad E. Sleebs[1,2*], Robin B. Gasser[1*]

[1] *Department of Veterinary Biosciences, Melbourne Veterinary School, Faculty of Science, The University of Melbourne, Parkville, Victoria 3010, Australia*
[2] *Walter and Eliza Hall Institute of Medical Research, Parkville, Victoria 3052, Australia*
[3] *Melbourne Mass Spectrometry and Proteomics Facility, The Bio21 Molecular Science and Biotechnology Institute, The University of Melbourne, Parkville, Victoria 3010, Australia*
[4] *Medical Parasitology and Infection Biology, Swiss Tropical and Public Health Institute, Allschwil, Switzerland*

Supplementary Methods – Chemistry experimental.

Supplementary Table S1. Cross-species *in vitro* activity of UMW-934 and selected analogues.

SUPPLEMENTARY METHODS

Chemistry experimental

General Chemistry Procedures. All non-aqueous reactions were performed under an atmosphere of nitrogen, unless otherwise specified. Commercially available reagents were used without further purification. Flash chromatography was performed with silica gel 60 (particle size 0.040–0.063 µm) on a CombiFlash Rf Purification System (Teledyne Isco) with mobile phase gradients as specified. NMR spectra were recorded on a Bruker Avance DRX 300 with the solvents indicated (^{1}H NMR at 300 MHz). Chemical shifts are reported in ppm on the δ scale and referenced to the appropriate solvent peak. LCMS were analyzed on an Agilent LCMS system equipped with an Agilent G6120B Mass Detector, 1260 Infinity G1312B Binary pump, 1260 Infinity G1367E HiPALS autosampler, and 1260 Infinity G4212B Diode Array Detector. The LCMS conditions were as follows: column: Luna Omega (1.6 µm, C18, 50 x 2.1 mm); injection volume: 1 µL; gradient: 5-100% B over 3.8 min (solvent A: water/0.1% formic acid; solvent B: ACN/ 0.1% formic acid); acquisition time: 4.1 min; flow rate: 1 mL/min; detection: 254 and 214 nm. Unless otherwise noted, all compounds were found to be >95% pure by this method. The preparative LCMS purification were performed using a Waters preparative HPLC system equipped with Waters ZQ 3100 –Mass Detector, Waters 2545-Pump, Waters SFO System Fluidics Organizer, Waters 2996 Diode Array Detector and Waters 2767 Sample Manager. The preparative-HPLC conditions were as follows: XBridge BEH C18 OBD Prep Column (130Å, 5 µm, 19 mm x 100 mm); injection volume: 1 mL; gradient is variable over 20 min depending on each compound (solvent A: water/0.1% formic acid; solvent B: ACN/ 0.1% formic acid); flow rate, 20 mL/min; detection 100 to 600 nm. HRMS were acquired through the Bio21 Mass Spectrometry and Proteomics Facility using a Thermo Scientific nano-LC Q Exactive Plus Mass spectrometer.

Compounds **2–6** were procured from commercial vendors and their purity was confirmed by LCMS.

Synthesis of 4-(5-Propylpyridin-2-yl)benzonitrile UMW-934

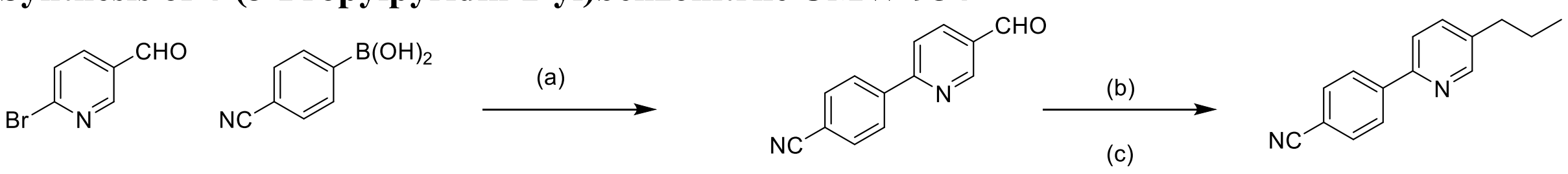

1 **2** **3** **4**

Scheme 1. *Reaction conditions*: (a) Pd(dppf)Cl_2.CH_2Cl_2, K_2CO_3, 1,4-dioxane/H_2O (9:1 ratio), 110 ºC; (b) $EtPPh_3Br$, *t*BuOK, THF, r.t; (c) 10% Pd/C, H_2, MeOH, r.t.

General procedure A: A mixture of aryl halide **int 1** (1 eq.), boronic acid **int 2** (1.1 eq.) and K_2CO_3 (2.5 eq.) in 1,4-dioxane/H_2O (9:1 ratio) was stirred at r.t and purged with N_2. To this mixture, Pd(dppf)Cl_2.CH_2Cl_2 (0.05 eq.) was added and then the reaction mixture was heated at 110 ºC (monitored by LCMS). The reaction mixture was filtered through a Celite pad and concentrated. The crude product was purified by flash column chromatography, eluting with 0-20% EtOAc/heptane to give the bi-aryl compound.

4-(5-Formylpyridin-2-yl)benzonitrile int 3
General procedure **A** was followed using 6-bromopyridine-3-carbaldehyde **int 1** (1 g, 5.4 mmol) and (4-cyanophenyl)boronic acid **int 2** (0.87 g, 5.9 mmol). Yield 83%, 0.93 g; beige solid. LC-MS (ESI) t_R 1.82 min, *m*/*z* 209.2 (M+H)$^+$. ^{1}H NMR (300 MHz, $CDCl_3$) δ 10.18 (s, 1H), 9.18 (s, 1H), 8.30 (d, *J* 6.9 Hz, 1H), 8.22 (d, *J* 8.3 Hz, 2H), 7.96 (d, *J* 8.3 Hz, 1H), 7.82 (d, *J* 8.2 Hz, 2H). The ^{1}H-NMR spectrum matches previous reported data. *Journal of Organic Chemistry (2001), 66(4), 1500-1502*

4-(5-Propylpyridin-2-yl)benzonitrile 1 (UMW-934)
To a stirred solution of ethyltriphenylphosphonium bromide (1.07 g, 2.88 mmol, 1.2 eq.) in THF (20 mL) was added *t*-BuOK (0.33 g, 2.9 mmol, 1.2 eq.). The reaction mixture was then stirred at r.t for 30 min. The 4-(5-formyl-2-pyridyl)benzonitrile **int 3** (0.50 g, 2.4 mmol, 1 eq.) was then added in three portions and the reaction was stirred at r.t for 4 h (monitored by LCMS). The mixture was filtered through a Celite pad and concentrated. The crude product was purified by flash column chromatography, eluting with 0-10% EtOAc/heptane to give the alkene intermediate (330 mg, 62% yield). LC-MS (ESI) t_R 2.30 min, *m*/*z* 221.2 (M+H)$^+$. This alkene

intermediate was dissolved in MeOH (20 mL) and 10% Pd/C was added. The reaction solution was purged with H_2 (3x) and left to stir at r.t under H_2 atmosphere until the starting material was fully converted to the product (monitored by LCMS). The reaction solution was filtered through a Celite pad and concentrated. The crude product was purified by flash column chromatography, eluting with 0-10% EtOAc/heptane to give compound **1** (300 mg, 56% yield over 2 steps). LC-MS (ESI) t_R 2.21 min, *m/z* 223.2 $(M+H)^+$. ^{1}H NMR (300 MHz, $CDCl_3$) δ 8.58 (d, *J* 2.2 Hz, 1H), 8.13 (d, *J* 8.5 Hz, 2H), 7.78 (d, *J* 8.4 Hz, 2H), 7.72 (dd, *J* 8.1, 0.9 Hz, 1H), 7.64 (dd, *J* 8.1, 2.3 Hz, 1H), 2.68 (d, *J* 7.7 Hz, 2H), 1.80 – 1.64 (m, 2H), 1.01 (t, *J* 7.3 Hz, 3H). ^{13}C NMR (75 MHz, $CDCl_3$) δ 152.7, 150.1, 143.3, 138.1, 137.4, 132.7, 127.4, 120.9, 119.1, 112.3, 34.9, 24.3, 13.8.

General Synthesis of O-substituted analogues

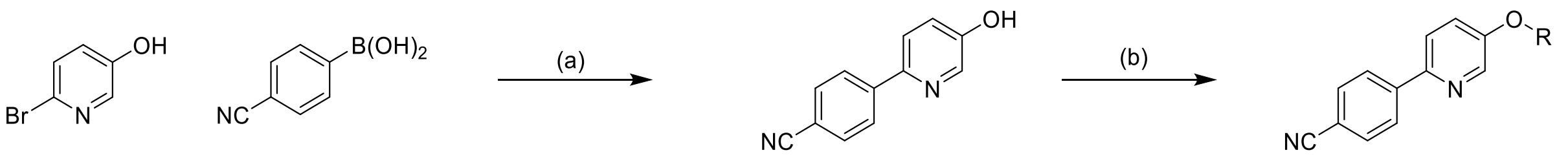

5 **2** **6** **7**

Scheme 2. *Reaction conditions*: (a) $Pd(dppf)Cl_2.CH_2Cl_2$, K_2CO_3, 1,4-dioxane/H_2O (9:1 ratio), 110 ºC; (b) R-I or R-Br, K_2CO_3, DMF, 90 ºC.

4-(5-Hydroxypyridin-2-yl)benzonitrile int 3

General procedure **A** was followed using 6-bromopyridin-3-ol **int 1** (1 g, 5.7 mmol) and (4-cyanophenyl)boronic acid **int 2** (1 g, 6.9 mmol). Yield 71%, 0.8 g; beige solid. LC-MS (ESI) t_R 1.49 min, *m/z* 197.2 $(M+H)^+$. ^{1}H NMR (300 MHz, DMSO) δ 10.32 (s, 1H), 8.27 (d, *J* 2.8 Hz, 1H), 8.17 (d, *J* 8.5 Hz, 2H), 7.94 (d, *J* 8.6 Hz, 1H), 7.88 (d, *J* 8.4 Hz, 2H), 7.28 (dd, *J* 8.7, 2.9 Hz, 1H). ^{13}C NMR (75 MHz, DMSO) δ 154.1, 144.9, 143.1, 138.2, 132.6, 126.3, 123.0, 121.9, 119.1, 110.1.

4-(5-Ethoxypyridin-2-yl)benzonitrile 7

General procedure **B** was followed using **int 3** (50 mg, 0.25 mmol) and iodoethane (31 uL, 0.38mmol). Yield 73%, 42 mg; colourless solid. LC-MS (ESI) t_R 2.29 min, *m/z* 225.2 $(M+H)^+$. ^{1}H NMR (300 MHz, $CDCl_3$) δ 8.43 (d, *J* 2.9 Hz, 1H), 8.09 (d, *J* 8.4 Hz, 2H), 7.84 – 7.69 (m, 3H), 7.33 (d, *J* 8.4 Hz, 1H), 4.18 (q, *J* 7.0 Hz, 2H), 1.50 (t, *J* 7.0 Hz, 3H). ^{13}C NMR (75 MHz, $CDCl_3$) δ 155.3, 147.2, 137.3, 133.0, 132.6, 126.9, 122.6, 121.7, 118.9, 111.9, 64.4, 14.7. HRMS *m/z*: $[M+H]^+$ Calcd for $C_{14}H_{12}N_2O$ 225.1022; Found 225.1020.

4-(5-Propoxypyridin-2-yl)benzonitrile 8 (WEHI-614)

General procedure **B** was followed using **int 3** (50 mg, 0.25 mmol) and 1-iodopropane (37 µL, 0.38 mmol). Yield 38%, 23 mg; beige solid. LC-MS (ESI) t_R 2.36 min, *m/z* 239.2 $(M+H)^+$. ^{1}H NMR (300 MHz, $CDCl_3$) δ 8.43 (d, *J* 2.9 Hz, 1H), 8.11 – 8.04 (m, 2H), 7.79 – 7.70 (m, 3H), 7.34 – 7.28 (m, 1H), 4.05 (t, *J* 6.5 Hz, 2H), 1.88 (p, *J* 7.0 Hz, 2H), 1.10 (t, *J* 7.4 Hz, 3H). ^{13}C NMR (75 MHz, $CDCl_3$) δ 155.4, 147.2, 143.0, 138.1, 132.6, 126.8, 121.9, 121.4, 119.1, 111.6, 70.2, 22.5, 10.4. HRMS *m/z*: $[M+H]^+$ Calcd for $C_{15}H_{14}N_2O$ 239.1179; Found 239.1177.

4-(5-Butoxypyridin-2-yl)benzonitrile 9

General procedure **B** was followed using **int 3** (50 mg, 0.25 mmol) and 4-bromobut-1-ene (39 µL, 0.38 mmol) to give the alkene intermediate. LC-MS (ESI) t_R 2.55 min, *m/z* 251.2 $(M+H)^+$. The alkene intermediate was dissolved in MeOH (5 mL) and then 10% Pd/C (0.4 mg) was added. The reaction solution was purged with H_2 (3 x) and then stirred under H_2 atmosphere for 2 h (monitored by LCMS). The solvent was removed and the crude product was purified by flash column chromatography, eluting with 0-20% EtOAc/heptane to give the title compound (38 mg, 59% yield over 2 steps) as a colourless solid. LC-MS (ESI) t_R 2.74 min, *m/z* 253.2 $(M+H)^+$. ^{1}H NMR (300 MHz, $CDCl_3$) δ 8.40 (d, *J* 2.9 Hz, 1H), 8.05 (d, *J* 8.3 Hz, 2H), 7.75 – 7.66 (m, 3H), 7.31 – 7.25 (m, 1H), 4.07 (t, *J* 6.5 Hz, 2H), 1.90 – 1.75 (m, 2H), 1.59 – 1.45 (m, 2H), 1.00 (t, *J* 7.4 Hz, 3H). ^{13}C NMR (75 MHz, $CDCl_3$) δ 155.5, 147.3, 143.2, 138.1, 132.7, 126.9, 122.0, 121.6, 119.1, 111.7, 68.5, 31.3, 19.3, 13.9. HRMS *m/z*: $[M+H]^+$ Calcd for $C_{16}H_{16}N_2O$ 253.1335; Found 253.1337.

4-(5-Isobutoxypyridin-2-yl)benzonitrile 10
General procedure **B** was followed using **int 3** (50 mg, 0.25 mmol) and 1-bromo-2-methyl-propane (41 µL, 0.38 mmol). Yield 65%, 42 mg; colourless solid. LC-MS (ESI) t_R 2.73 min, *m/z* 253.2 $(M+H)^+$. ^{1}H NMR (300 MHz, $CDCl_3$) δ 8.43 (d, *J* 2.9 Hz, 1H), 8.12 – 8.05 (m, 2H), 7.79 – 7.69 (m, 3H), 7.35 – 7.26 (m, 1H), 3.85 (d, *J* 6.5 Hz, 2H), 2.26 – 2.09 (m, 1H), 1.09 (d, *J* 6.7 Hz, 6H). ^{13}C NMR (75 MHz, $CDCl_3$) δ 155.4, 147.3, 143.3, 138.3, 132.5, 126.8, 121.6, 121.3, 119.0, 111.5, 74.9, 28.3, 19.2. HRMS *m/z*: $[M+H]^+$ Calcd for $C_{16}H_{16}N_2O$ 253.1335; Found 253.1336.

4-(5-(*sec*-Butoxy)pyridin-2-yl)benzonitrile 11
General procedure **B** was followed using **int 3** (50 mg, 0.25 mmol) and 2-bromobutane (41 µL, 0.38 mmol). Yield 63%, 41 mg; colourless solid. LC-MS (ESI) t_R 2.65 min, *m/z* 253.2 $(M+H)^+$. ^{1}H NMR (300 MHz, $CDCl_3$) δ 8.41 (dd, *J* 3.0, 0.6 Hz, 1H), 8.11 – 8.04 (m, 2H), 7.78 – 7.69 (m, 3H), 7.33 – 7.26 (m, 1H), 4.54 – 4.37 (m, 1H), 1.96 – 1.66 (m, 2H), 1.38 (d, *J* 6.1 Hz, 3H), 1.03 (t, *J* 7.4 Hz, 3H). ^{13}C NMR (75 MHz, $CDCl_3$) δ 154.6, 147.2, 143.4, 139.5, 132.6, 126.8, 122.9, 121.4, 119.1, 111.5, 76.1, 29.1, 19.2, 9.7. HRMS *m/z*: $[M+H]^+$ Calcd for $C_{16}H_{16}N_2O$ 253.1335; Found 253.1338.

4-(5-(Isopentyloxy)pyridin-2-yl)benzonitrile 12
General procedure **B** was followed using **int 3** (50 mg, 0.25 mmol) and 1-bromo-3-methyl-butane (46 µL, 0.38 mmol). Yield 55%, 37 mg; colourless solid. LC-MS (ESI) t_R 2.90 min, *m/z* 267.2 $(M+H)^+$. ^{1}H NMR (300 MHz, $CDCl_3$) δ 8.42 (d, *J* 3.5 Hz, 1H), 8.11 – 8.05 (m, 2H), 7.78 – 7.70 (m, 3H), 7.34 – 7.28 (m, 1H), 4.12 (t, *J* 6.6 Hz, 2H), 1.98 – 1.82 (m, 1H), 1.75 (q, *J* 6.7 Hz, 2H), 1.01 (d, *J* 6.6 Hz, 6H). ^{13}C NMR (75 MHz, $CDCl_3$) δ 155.4, 147.4, 143.4, 138.4, 132.7, 126.9, 121.7, 121.5, 119.2, 111.6, 67.2, 37.9, 25.1, 22.7. HRMS *m/z*: $[M+H]^+$ Calcd for $C_{17}H_{18}N_2O$ 267.1492; Found 267.1491.

4-(5-(Cyclopentyloxy)pyridin-2-yl)benzonitrile 13
General procedure **B** was followed using **int 3** (50 mg, 0.25 mmol) and 1-bromo-3-methyl-butane (41 µL, 0.38 mmol). Yield 59%, 40 mg; colourless solid. LC-MS (ESI) t_R 2.77 min, *m/z* 265.2 $(M+H)^+$. ^{1}H NMR (300 MHz, $CDCl_3$) δ 8.39 (d, *J* 2.9 Hz, 1H), 8.11 – 8.04 (m, 2H), 7.79 – 7.69 (m, 3H), 7.32 – 7.25 (m, 2H), 4.95 – 4.84 (m, 1H), 2.12 – 1.79 (m, 6H), 1.79 – 1.63 (m, 2H). ^{13}C NMR (75 MHz, $CDCl_3$) δ 154.4, 147.1, 143.4, 139.1, 132.6, 126.8, 122.7, 121.4, 119.2, 111.5, 80.2, 32.9, 24.2. HRMS *m/z*: $[M+H]^+$ Calcd for $C_{17}H_{16}N_2O$ 265.1335; Found 265.1333.

4-(5-(Cyclobutylmethoxy)pyridin-2-yl)benzonitrile 14
General procedure **B** was followed using **int 3** (50 mg, 0.25 mmol) and bromomethylcyclobutane (43 µL, 0.38 mmol). Yield 67%, 45 mg; beige solid. LC-MS (ESI) t_R 2.81 min, *m/z* 265.2 $(M+H)^+$. ^{1}H NMR (300 MHz, $CDCl_3$) δ 8.42 (d, *J* 2.9 Hz, 1H), 8.07 (d, *J* 8.5 Hz, 2H), 7.79 – 7.69 (m, 3H), 7.30 (dd, *J* 9.6, 2.0 Hz, 1H), 4.05 (d, *J* 6.6 Hz, 2H), 2.93 – 2.75 (m, 1H), 2.26 – 2.12 (m, 2H), 2.11 – 1.83 (m, 4H). ^{13}C NMR (75 MHz, $CDCl_3$) δ 155.5, 147.4, 143.4, 138.4, 132.6, 126.9, 121.8, 121.4, 119.1, 111.5, 72.9, 34.6, 24.9, 18.9. HRMS *m/z*: $[M+H]^+$ Calcd for $C_{17}H_{16}N_2O$ 265.1335; Found 265.1337.

4-(5-(Cyclohexylmethoxy)pyridin-2-yl)benzonitrile 15 (WEHI-684)
General procedure **B** was followed using **int 3** (50 mg, 0.25 mmol) and bromomethylcyclohexane (68 mg, 0.38 mmol). Yield 64%, 48 mg; colourless solid. LC-MS (ESI) t_R 2.95 min, *m/z* 293.2 $(M+H)^+$. ^{1}H NMR (300 MHz, $CDCl_3$) δ 8.42 (d, *J* = 2.9 Hz, 1H), 8.11 – 8.04 (m, 2H), 7.79 – 7.69 (m, 3H), 7.34 – 7.26 (m, 1H), 3.88 (d, *J* 6.1 Hz, 2H), 2.07 – 1.59 (m, 7H), 1.47 – 0.95 (m, 6H). ^{13}C NMR (75 MHz, $CDCl_3$) δ 155.5, 147.3, 143.3, 138.3, 132.5, 126.8, 121.6, 121.3, 119.1, 111.4, 74.0, 37.6, 29.8, 26.4, 25.8. HRMS *m/z*: $[M+H]^+$ Calcd for $C_{19}H_{20}N_2O$ 293.1648; Found 293.1645.

4-(5-(Oxetan-3-ylmethoxy)pyridin-2-yl)benzonitrile 6
General procedure **B** was followed using **int 3** (50 mg, 0.25 mmol) and 3-(bromomethyl)oxetane (58 mg, 0.38 mmol). Yield 46%, 31 mg; colourless solid. LC-MS (ESI) t_R 1.95 min, *m/z* 267.2 $(M+H)^+$. ^{1}H NMR (300 MHz, $CDCl_3$) δ 8.44 (d, *J* 2.9 Hz, 1H), 8.11 – 8.04 (m, 2H), 7.75 (d, *J* 8.7 Hz, 3H), 7.37 (dd, *J* 8.7, 3.0 Hz, 1H), 4.92 (dd, *J* 7.8, 6.3 Hz, 2H), 4.60 (t, *J* 6.1 Hz, 2H), 4.33 (d, *J* 6.7 Hz, 2H), 3.58 – 3.42 (m, 1H). ^{13}C NMR (75 MHz, $CDCl_3$) δ 155.1, 147.9, 137.7, 132.6, 126.9, 122.2, 121.6, 118.9, 111.8, 74.0, 69.6, 34.5.

4-(5-Phenoxypyridin-2-yl)benzonitrile 17

A mixture of **int 3** (72 mg, 0.37 mmol), iodobenzene (27 µL, 0.25 mmol), CuI (0.47 mg, 0.0025 mmol), Cs_2CO_3 (170 mg, 0.51 mmol), and 2,2,6,6-tetramethylheptane-3,5-dione (TMHD) (4.5 mg, 0.025 mmol) in DMF (1 mL) was heated at 60 °C for 48 h. The heterogeneous mixture was cooled to r.t, diluted with ethyl acetate and filtered through a pad of Celite. The solvent was removed and the crude product was purified by flash column chromatography, eluting with 0-20% EtOAc/heptane to give **17** (29 mg, 43% yield) as a colourless solid. LC-MS (ESI) t_R 2.71 min, *m*/*z* 273.2 $(M+H)^+$. ^1H NMR (300 MHz, $CDCl_3$) δ 8.53 (dd, *J* 2.9, 0.7 Hz, 1H), 8.14 – 8.08 (m, 2H), 7.81 – 7.73 (m, 3H), 7.49 – 7.38 (m, 3H), 7.27 – 7.20 (m, 1H), 7.11 (dd, *J* 8.5, 1.2 Hz, 2H). ^{13}C NMR (75 MHz, $CDCl_3$) δ 156.1, 154.3, 149.8, 143.1, 141.3, 132.7, 130.3, 127.2, 126.1, 124.7, 121.6, 119.5, 119.1, 112.3.

Supplementary Table S1. Cross-species *in vitro* activity of UMW-934 and selected analogues. The *in vitro* activity of UMW-934 and selected analogues was evaluated against multiple developmental stages of parasitic helminths representing nematodes and a trematode using established methods.[64] Larval or adult worms were incubated with individual compounds for 72 h under species-specific culture conditions. Compounds were tested at a final concentration of 10 µM. For *Heligmosomoides polygyrus*, third-stage larvae (L3) and adults were assessed at 50 µM. Worm death is expressed as percentage lethality relative to vehicle (DMSO) controls and represents the mean ± standard deviation (SD) from triplicate measurements. For larval stages, death was determined by the absence of motility following stimulation with hot water (~80 °C). For adult worms, viability was assessed using a standardised motility scoring system. A dash (–) indicates that the stage was not assessed for that compound. *Abbreviations:* L3, ensheathed third-stage larvae; Adults, adult worms; NTS, newly transformed schistosomula.

Compound	*Ancylostoma ceylanicum* L3	*Necator americanus* L3	*Heligmosomoides polygyrus* L3	*Heligmosomoides polygyrus* Adult	*Strongyloides ratti* L3	*Trichuris muris* Adult	*Schistosoma mansoni* NTS
UMW-934 (1)	28.3±4.1	2.5±4.6	25.5±6.2	–	0.0±0.0	31.3±6.3	38.8±1.3
4	38.2±14.2	3.4±3.6	100.0±0.0	100.0±0.0	2.3±1.9	43.8±6.3	30.5±5.5
7	10.6±0.8	0.4±7.5	38.2±4.8	–	35.6±11.0	14.6±2.1	34.6±2.8
9	31.1±3.5	20.1±6.6	37.2±6.0	–	2.1±1.3	25.0±0.0	26.5±1.5
14	17.2±4.0	8.3±0.5	42.8±3.0	–	3.7±1.7	43.8±6.3	32.6±0.7
10	12.1±3.2	17.9±4.4	96.8±3.3	100.0±0.0	35.5±7.4	45.8±0.0	34.6±5.4
11	38.1±6.7	4.0±2.7	38.5±1.5	–	15.4±4.2	50.0±0.0	43.0±7.0
12	21.3±6.6	22.2±7.0	37.9±7.8	–	24.4±7.8	25.0±0.0	36.7±3.3
WEHI-684 (15)	17.2±2.8	12.2±6.6	14.2±6.7	–	16.3±11.3	50.0±0.0	28.6±0.6
17	27.2±7.0	14.3±2.9	59.8±4.8	–	24.4±12.5	39.6±2.1	26.5±1.5
